# Climate Warming as a Possible Trigger of Keystone Mussel Population Decline in Oligotrophic Rivers at the Continental Scale

**DOI:** 10.1038/s41598-017-18873-y

**Published:** 2018-01-08

**Authors:** Ivan N. Bolotov, Alexander A. Makhrov, Mikhail Yu. Gofarov, Olga V. Aksenova, Paul E. Aspholm, Yulia V. Bespalaya, Mikhail B. Kabakov, Yulia S. Kolosova, Alexander V. Kondakov, Thomas Ofenböck, Andrew N. Ostrovsky, Igor Yu. Popov, Ted von Proschwitz, Mudīte Rudzīte, Māris Rudzītis, Svetlana E. Sokolova, Ilmari Valovirta, Ilya V. Vikhrev, Maxim V. Vinarski, Alexey A. Zotin

**Affiliations:** 1 Laboratory for Evolutionary Ecology and Phylogenetics, Northern Arctic Federal University, Arkhangelsk, Russia; 2 Institute of Biogeography and Genetic Resources, Federal Center for Integrated Arctic Research, Russian Academy of Sciences, Arkhangelsk, Russia; 3 Laboratory for Ecology of Aquatic Communities and Invasions, A.N. Severtsov Institute of Problems of Ecology and Evolution, Russian Academy of Sciences, Moscow, Russia; 4 Department of Natural Resources and Rural Development, Norwegian Institute of Bioeconomy Research, Svanhovd, Svanvik, Norway; 5 Municipal Department 45 – Water Management, Vienna City Administration, Vienna, Austria; 6 Department of Invertebrate Zoology, Saint Petersburg State University, Saint Petersburg, Russia; 7 Department of Palaeontology, Geozentrum, University of Vienna, Vienna, Austria; 8 Department of Applied Ecology, Saint Petersburg State University, Saint Petersburg, Russia; 9 Section of Invertebrate Zoology, Göteborg Natural History Museum, Göteborg, Sweden; 10 Museum of History of Science and Technology, University of Latvia, Riga, Latvia; 11 Finnish Museum of Natural History, University of Helsinki, Helsinki, Finland; 12 Laboratory of Macroecology and Biogeography of Invertebrates, Saint Petersburg State University, Saint Petersburg, Russia; 13 Laboratory of Evolutionary Biology of Development, N.K. Koltzov Institute of Developmental Biology of Russian Academy of Sciences, Moscow, Russia

## Abstract

The effects of climate change on oligotrophic rivers and their communities are almost unknown, albeit these ecosystems are the primary habitat of the critically endangered freshwater pearl mussel and its host fishes, salmonids. The distribution and abundance of pearl mussels have drastically decreased throughout Europe over the last century, particularly within the southern part of the range, but causes of this wide-scale extinction process are unclear. Here we estimate the effects of climate change on pearl mussels based on historical and recent samples from 50 rivers and 6 countries across Europe. We found that the shell convexity may be considered an indicator of the thermal effects on pearl mussel populations under warming climate because it reflects shifts in summer temperatures and is significantly different in viable and declining populations. Spatial and temporal modeling of the relationship between shell convexity and population status show that global climate change could have accelerated the population decline of pearl mussels over the last 100 years through rapidly decreasing suitable distribution areas. Simulation predicts future warming-induced range reduction, particularly in southern regions. These results highlight the importance of large-scale studies of keystone species, which can underscore the hidden effects of climate warming on freshwater ecosystems.

## Introduction

Many ecosystems are threatened because of climate warming, which affects the status of populations of individual species, their ecological functions and interspecific interactions^1–4^. The ranges of many terrestrial species are shifting rapidly in latitude or altitude in response to changing climate^5^. Inland surface waters are also affected by climate warming because of the close relationship between air temperature and surface water temperature^6–8^. However, predictions of freshwater biodiversity responses to climate change are difficult because detected range shifts are based on records from a small number of taxa from few regions^9^. Modeling processes in key aquatic habitats based on the identification and quantification of factors that control the distribution of biodiversity is therefore a topic of great importance^2,4,10^.

Current knowledge of recent warming in rivers is scarce due to a lack of long-term and large-scale studies in Europe as well as globally. However, a rapid rise in water temperature in European lotic systems in recent decades has been confirmed^8^. Recent studies reveal that among hydro-climatological variables, change in air temperature, which is a response to climate forcing, is the main driver of river temperature change because it had the highest correlation with river temperature irrespective of period^8,11^. The strongest impacts of warming water appear to be on cold-water ecosystems, such as oligotrophic streams and rivers. Oligotrophic rivers are scattered throughout Europe and are mostly concentrated in the northern region of the continent and locally present within mountain systems^12^. These rivers harbor unique and vulnerable communities with several keystone species, including salmonid fishes and freshwater pearl mussels^10,13,14^. Changes in the populations of keystone species have the greatest effect because of their critical role in ensuring the functioning of natural ecosystems^15,16^. Unfortunately, the effects of climate change on oligotrophic rivers and their communities are investigated only at local and regional scales^17–20^.

**Figure 1:**
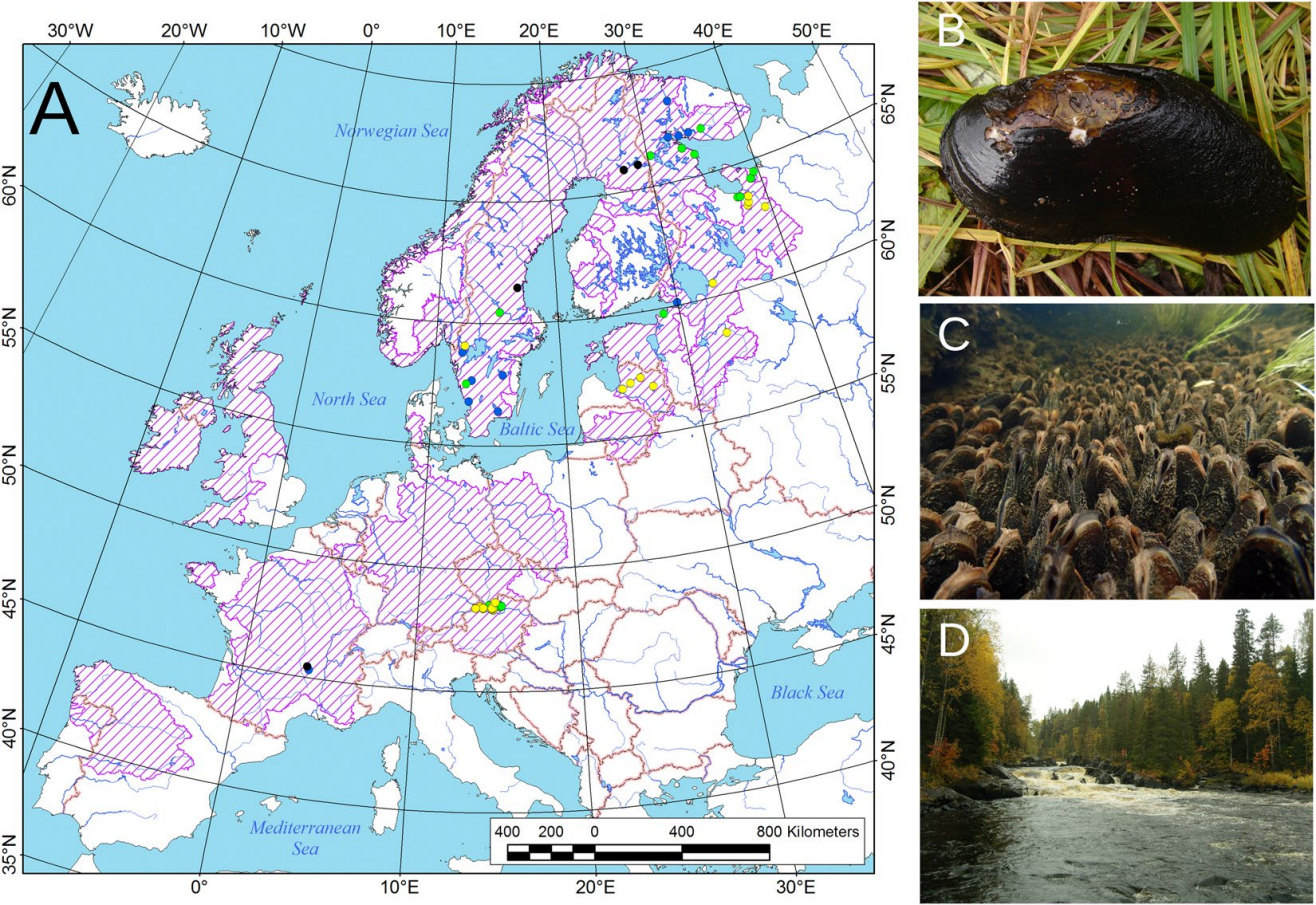
European distribution range of *Margaritifera margaritifera* with our sampling localities, adult mussels and their habitat. (**A**) Map of the distribution range of the freshwater pearl mussel in Europe and our sampling localities. The pink hatching indicates the approximate distribution range of the species^21^. Blue dots indicate historical samples (~1840–1940; *n* = 13); yellow dots indicate recent declining populations (1984–2013; *n* = 24); green dots indicate recent viable populations (1984–2013; *n* = 21); and black dots indicate recent populations with unknown status (1984–2013; *n* = 4). Map was performed by using ESRI ArcGIS 10 software (www.esri.com/arcgis). The base of the map was created with Natural Earth Free Vector and Raster Map Data (www.naturalearthdata.com). (**B**) A mussel specimen (photo: Oleg N. Bespaliy). (**C**) Undisturbed mussel bed, Finland (photo: Panu Oulasvirta). (**D**) Habitat, NW Russia (photo: Olga V. Aksenova).

Our study species is the freshwater pearl mussel, *Margaritifera margaritifera* (L., 1758) (Fig. 1A–D), which is among the most critically endangered freshwater animals at the global scale^16,21^. This species exclusively inhabits cold running waters with low mineralization and organic content and is unique because of its very high longevity (up to 280 years) and narrow host specialization^6,21,22^. The distribution and abundance of *M. margaritifera* have drastically decreased throughout Europe over the last century, particularly within the southern part of the range^23–25^ and the majority of its populations has lacked successful reproduction for last 30–50 years^16,26^. The indirect effects associated with anthropogenic transformations, such as habitat degradation, alteration and fragmentation as well as salmonid host overfishing, are considered the most important factors for the decline of the species^16,17,27^. The nitrate concentration positively correlated with mortality rates of the adult pearl mussels, while phosphate, calcium and BOD_5_ correlated with decreasing juvenile survival^22^. Progressive eutrophication of oligotrophic water bodies may also affect salmonid hosts, with an inhibition of their natural reproduction^28^. However, little attention has been paid to global climate change as a possible factor in population decline^17–19^.

Under this gap of knowledge, the present study aims to: (i) perform a series of regression models describing the influence of climatic variables on the shell convexity, age and status of freshwater pearl mussel populations at the continental scale; (ii) produce a series of temperature dependent integrative models of climatically suitable areas for freshwater pearl mussels throughout Europe during past and future periods on the basis of morphometric, ecological and climatic data; (iii) discuss the possible influence of climate warming on freshwater pearl mussel population decline during the last century. Based on spatial and temporal modeling of the relationship between shell convexity and population status, we determined that global climate change could have accelerated the population decline of freshwater pearl mussels over the last 100 years through rapidly decreasing suitable distribution areas.

## Results

### Shell convexity changes in historical and recent *M. margaritifera* populations

**Figure 2:**
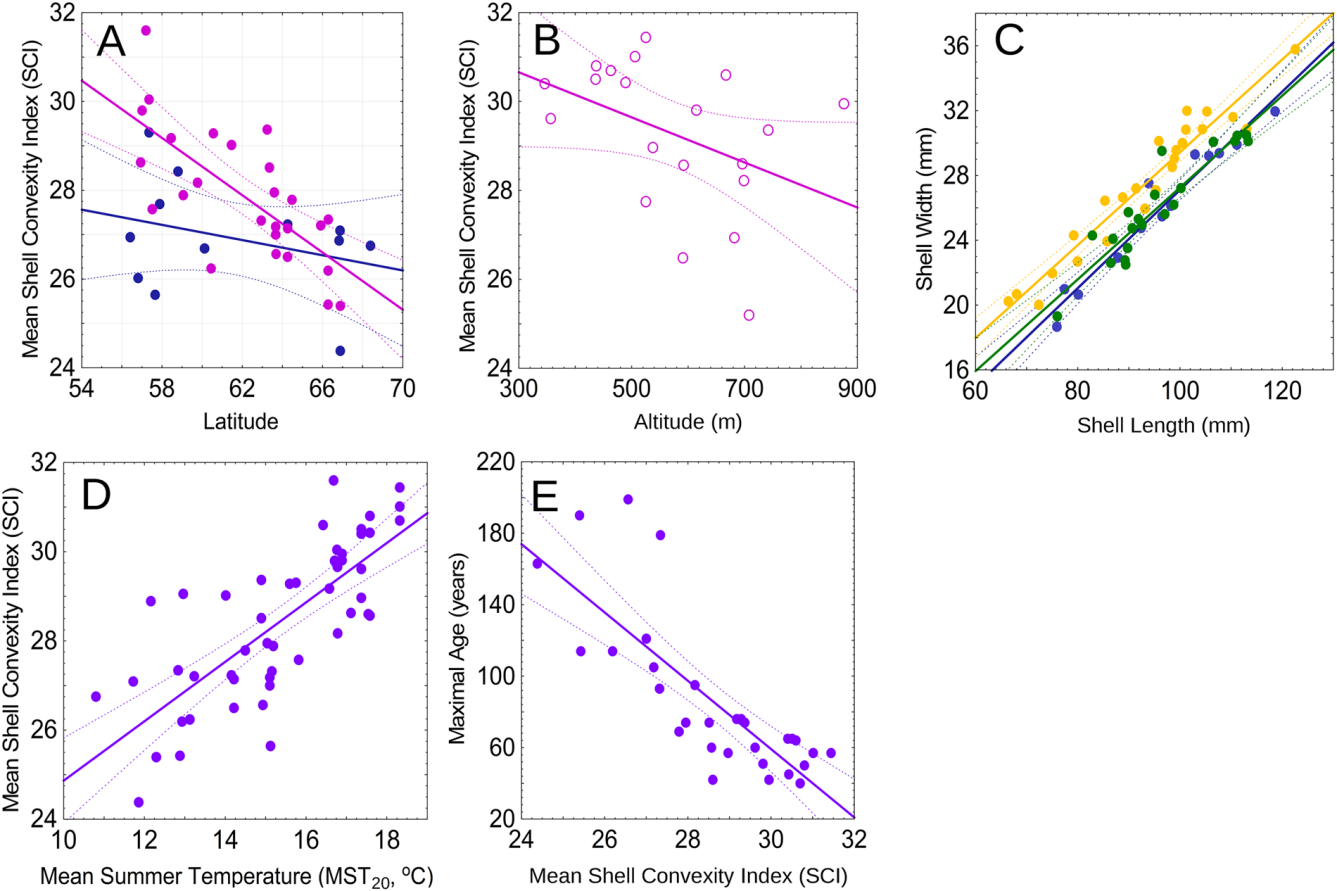
Variability of the relative shell convexity (SCI) and maximum age of *Margaritifera margaritifera* populations. The dashed lines are the 95% confidence bounds of the regression models. (**A**) Latitude vs. mean SCI scatterplot in populations from lowland rivers across Europe (<200 m altitude). Each point represents the mean value in a population; red points are recent lowland populations (1984–2013, *n* = 25, significant latitudinal trend: Spearman’s rank correlation, *P* = 0.000001), and blue points are historical lowland populations (~1840–1940, *n* = 12, no significant latitudinal trend: Spearman’s rank correlation, *P* = 0.53). (**B**) Altitude vs. mean SCI scatterplot in recent populations from mountainous rivers in Austria (>300 m altitude). Each point represents the mean value in a population (1992, *n* = 20, significant altitudinal trend: Spearman’s rank correlation, *P* = 0.02). (**C**) Scatterplot of population shell length vs. shell width. Each point represents the mean value in a population; green points are recent viable populations (1984–2013, *n* = 21), yellow points are recent declining populations (1984–2013, *n* = 24), and blue points are historical populations (~1840–1940, *n* = 13). (**D**) Scatterplot of mean summer temperature (MST_20_, 20-year mean before sampling) vs. mean SCI (equation 1). (**E**) Scatterplot of mean SCI vs. maximum age (equation 2). Each point represents the mean value in a population (*n* = 49).

Here we report the results of tests of the climate change effect on freshwater pearl mussel populations based on extensive morphological data sets obtained from 50 rivers and 6 countries across Europe: Austria, Latvia, Finland, France, Russia and Sweden (Supplementary Tables 1 and 2). The mean shell convexity index (SCI = width/length ratio × 100) in historical pearl mussel samples from lowland rivers (~1840–1940) does not reveal a significant latitudinal trend (Spearman’s rank correlation between latitude and SCI: *r*_s_ = −0.20 (n.s.), *n* = 12, *P* = 0.53) (Fig. 2A). In contrast, this index in recent samples from lowland rivers (1984–2013) increases from high to low latitudes (Spearman’s rank correlation between latitude and SCI: *r*_s_ = −0.80, *n* = 25, *P* = 0.000001) (Fig. 2A). The separate-slopes model indicates that latitude has different effects on the mean SCI at different (historical and recent) time intervals (*F* = 14.1, *df* = 2, *P* < 0.001; see Supplementary Table 3 for details). However, the confidence intervals of regression lines in historical and recent samples overlap over a range at high latitudes between 61 and 70°N. This pattern suggests that the significant latitudinal trend in recent samples is mainly associated with a continual SCI shift in populations south of 61°N, whereas in the northernmost populations this shift cannot be traced. Additionally, the mean SCI in recent samples from mountain rivers at the regional scale (Austria) increases from high to low altitudes (Spearman’s rank correlation between latitude and SCI: *r*_s_ = −0.51, *n* = 20, *P* = 0.02) (Fig. 2B).

Results of general linear models (GLMs) revealed that in recent populations the relative shell width is significantly influenced by the population status independently of shell size; i.e., the mean SCI in recent declining populations differs from that for recent viable populations (Table 1). Furthermore, based on GLMs, the relative shell width does not differ between recent viable populations and historical samples, but differs significantly between recent declining populations and historical samples (Table 1). The mean SCI value in recent declining populations is significantly higher than those in recent viable populations (Fig. 2C; Student’s t-test: *t* = 6.3, *df* = 43, *P* < 0.0001) and in historical samples (Fig. 2C; Student’s t-test: *t* = 6.5, *df* = 35, *P* < 0.0001). This parameter does not show differences between recent viable populations and historical samples (Fig. 2C; Student’s t-test: *t* = 0.7, *df* = 32; *P* = 0.5).

**Table 1:** Results of general linear models (GLMs) of shell width in recent (1984–2013) and historical (~1840–1940) populations of *Margaritifera margaritifera*.

**Response variable**	**Source**	**Selected population groups: (i) recent viable and (ii) declining (** ***R*** ^**2**^ ** = 0.91)**				**Selected population groups: (i) historical and (ii) recent viable (** ***R*** ^**2**^ ** = 0.91)**				**Selected population groups: (i) historical and (ii) recent declining (** ***R*** ^**2**^ ** = 0.94)**			
		***SS***	***d.f*** *.*	***F***	***P***	***SS***	***d.f*** *.*	***F***	***P***	***SS***	***d.f*** *.*	***F***	***P***
shell width	Intercept	—	—	—	n.s.	—	—	—	n.s.	—	—	—	n.s.
	length	32555.5	1	24740.6	<0.001	23308.6	1	18437.7	<0.001	24573.8	1	21576.3	<0.001
	Population group	51.9	1	39.4	<0.001	—	—	—	n.s.	47.6	1	41.8	<0.001
	Length × Population group	—	—	—	n.s.	—	—	—	n.s.	—	—	—	n.s.
	Error	56.6	43			41.7	33			39.9	35		

Regression models were simplified to the minimal adequate models.

### The impacts of climate change on the shell convexity in *M. margaritifera* populations

We discovered that the mean SCI values in *M. margaritifera* samples are directly associated with mean air summer temperature (Fig. 2D; linear regression of the mean SCI versus mean summer temperature during the 20-year period before shell collecting (MST_20_):

1 $${\rm{SCI}}=18.207+(0.666\times {{\rm{MST}}}_{20});{\rm{Pearson}}\,{\rm{r}}=0.76,{F}_{1,47}=64.2,P < 0.00001.$$

The k-fold cross-validation of the model (k = 5) revealed that the training models fit with the actual observations: mean absolute percentage deviation (MAPE) ≤5.5%, Tofallis’s relative accuracy measure ≤0.04, and min-max accuracy ≥0.95 (Supplementary Table 4). The training models are close to each other and to equation 1 (Supplementary Table 4). We found no significant spatial auto-correlation in the modeling SCI data set (observed Moran’s *I* = 0.51, expected Moran’s *I* = −0.02, variance = 0.48, *z*-score = 0.77, *p* = 0.44). Our testing with different periods of temperature averaging reveals that correlation with the mean SCI increases slightly with extension of the period but the coefficients under 20-, 30-, 40- and 50-year averaging were quite similar (Supplementary Table 5). We found that the MST is the best explanation for the observed variability in SCI in recent samples compared with mean temperature of spring, winter and autumn, annual mean temperature, monthly mean temperature, effective temperature sum (ETS) and thermal growing season length index (equation 1, Fig. 2D and Supplementary Fig. 3). Additionally, there is a highly negative correlation between the mean SCI and the maximum age in populations (Fig. 2E; linear regression of the maximum age versus mean SCI:

2 $${A}_{{\rm{\max }}}=632.846\,-\,(19.122\times {\rm{SCI}});{\rm{Pearson}}\,{r}=-0.80,{F}_{1,28}=48.7,P < 0.00001.$$

### Modeling of suitable areas for *M. margaritifera* under climate changes

**Figure 3:**
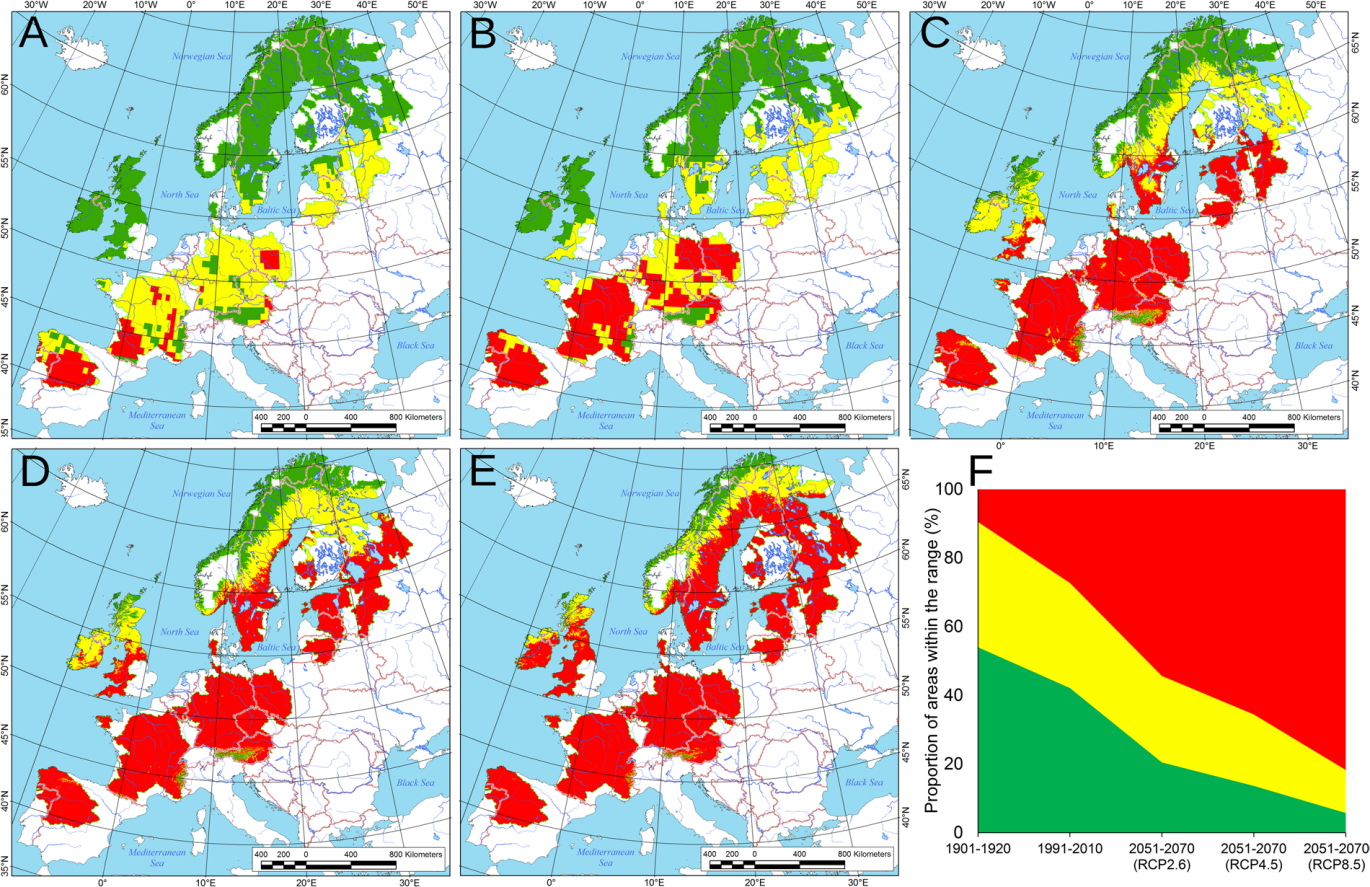
Spatial models of climatically suitable areas for freshwater pearl mussels across Europe in the past and future, based on equation 1 (see Methods section). Legend: green – suitable thermal conditions, viable populations; yellow – possible negative thermal effects, declining populations; red – most probably unsuitable areas. (**A**) Climatically suitable areas during the cold period from 1901–1920. (**B**) Climatically suitable areas during the warm period from 1991–2010. (**C**–**E**) Climatically suitable areas during the period from 2061–2080 under future climate change scenarios, low RCP 2.6 (**C**), moderate RCP 4.5 (**D**), and extreme RCP 8.5 (**E**). (**F**) Shift in climatically suitable areas based on spatial modeling. Areas are subdivided with respect to the prospective status of mussel populations: viable (green), declining (yellow), and under extinction or extinct (red). The climate change scenarios for 2051–2070 are as follows: low RCP 2.6, moderate RCP 4.5, and extreme RCP 8.5. Maps were performed by using ESRI ArcGIS 10 software (www.esri.com/arcgis). Climatic data sets were obtained from the CRU TS v. 3.23 climate database (Climatic Research Unit, University of East Anglia) and from the WorldClim v. 1.4 database. The base of the maps was created with Natural Earth Free Vector and Raster Map Data (www.naturalearthdata.com).

Reconstruction of long-term changes in the mean SCI in freshwater pearl mussel populations under climate fluctuations in the last 100 years (1901–2010) using equation 1 revealed the lowest index values at the beginning of the 20^th^ century and the maximum during the warm period of 1991–2010 (Supplementary Fig. 4). Correspondingly, reconstruction of maximum age changes using equation 2 indicated a significant decline in longevity during this time interval, particularly in recently declining populations (Supplementary Fig. 4). We developed a new approach to evaluate spatiotemporal shifts in climatically suitable areas for freshwater pearl mussels using equation 1 and the ranges of mean SCI in recent viable and declining populations. The 95% upper confidence boundary of the mean SCI in declining populations was used to delineate climatically unsuitable areas for the species. Spatial modeling revealed that suitable areas for *M. margaritifera* were widely present in Central and Southern Europe during the cold period of 1901–1920 (Fig. 3A). In contrast, these areas have been drastically reduced after climate warming during the period of 1991–2010 (Fig. 3B,F). Based on our models, currently viable populations largely remained in Northern Europe and in the northern part of the British Isles, but the majority of the former range appears to be unfavorable or even unsuitable for this species. The spatial models of climatically suitable areas during the period of 2061–2080 under low, moderate and extreme climate change scenarios (Fig. 3C–F) predict almost complete disappearance of freshwater pearl mussels across Central and Southern Europe.

## Discussion

### The mean SCI in freshwater pearl mussel populations as an indicator of climate changes

The SCI of freshwater mussels proved to be considerably influenced by allometric growth and therefore is not considered a suitable parameter for detecting trends in ecophenotypic variation^29^. However, it could be used for climatic reconstructions because growth patterns of the freshwater pearl mussels are significantly influenced by temperature with faster rates of shell growth in warmer environments (e.g., during warmer summers), likely because higher temperatures increase metabolic activity and hence rates of shell production^6,24,30,31^ (Supplementary Table 6). In general, the SCI reflects the temperature control of allometric growth that is expressed by shell convexity increasing with temperature via increased width of annual growth increments of the shell (Spearman’s rank correlation between mean growth coefficient (K) and SCI: r_s_ = 0.68, *n* = 9, *P* = 0.042).

Our analysis showed that SCI may be considered as a possible indicator of the thermal effects on *M. margaritifera* populations under warming climate conditions because it directly reflects shifts in mean summer temperatures and is significantly different in viable and declining populations. The effect of higher temperature on populations manifests itself in increased growth and metabolic rates, reduced longevity of adult mussels, high mortality of juveniles and restricted larval development (see Supplementary Table 6 for details). Warming climate may directly affect populations through high water temperatures and low dissolved oxygen levels but also could have indirect effects such as the eutrophication and so-called “browning” of rivers, increases in algae and macrophyte cover, massive floods and the depletion of host fish stocks^17–19,27,32^. The MST is the best explanation for the observed variability in SCI compared with other climatic variables that is consistent with studies on river warming because seasonal analyses showed that, while the rivers warmed in all seasons, the fastest warming occurred during summer^8^.

The three samples from the River Kamp system (Austria) revealed anomalous SCI values in relation to air temperature (Supplementary Fig. 2). The most probable cause for this artifact is a low-resolution climate model (0.5 × 0.5° grid squares), which does not consider local heterogeneity of thermal conditions within high-altitude regions. The significant correlation between altitude and SCI in Austrian rivers (see Fig. 2B) supports this suggestion. In mountainous areas, suitable conditions for freshwater pearl mussels may be locally preserved in certain cold streams and rivers such as in Kamp drainage basin, which harbors last remaining viable populations in Austria^26^. Similar observations were made for several mountain areas in Southern Europe (e.g., Portugal^19,25^).

### Possible warming-driven decline of freshwater pearl mussels throughout Europe

The results of our spatial modeling (Fig. 3A,B) correspond well to previously published data^16–18,21–26^ and indicate that the rapid decline of freshwater pearl mussel populations across Europe coincided with global climate change, which may increase multiple negative effects from local and regional anthropogenic transformation of freshwater ecosystems^16,17,27^. We suggest that cold climate periods contribute to optimal abiotic conditions for *M. margaritifera* as a keystone species and likely for oligotrophic river ecosystems as a whole. With respect to our model, the historical samples of *M. margaritifera* could be considered as representing viable populations that correspond to available data on the high abundance of populations throughout Europe during the 19^th^ century and the first half of the 20^th^ century^14,16,22,26^. Our modeling confirms a desirable environment for *M. margaritifera* populations in Europe at the beginning of 20^th^ century followed by rise of the populations’ decline due to climate warming later on.

Although our simplified temperature based model does not take into account many other environmental and human-mediated limiting factors (e.g., host fish decline, habitat degradation, and water pollution), the impact of which appears to be more significant in southern regions, this example of possible warming-driven species decline revealed that climate changes may have hidden effects on populations of individual species and on freshwater ecosystems. These effects may accelerate local climate-related extinctions, which are widespread among terrestrial animals, but are almost unknown in freshwater taxa^33^. Additionally, our findings highlight the role of high-altitude rivers as local but important refugia for cold-adapted freshwater species such as *M. margaritifera* under future climate warming scenarios. Finally, we revealed a new easy-to-obtain and low cost indicator of population status in freshwater pearl mussels (SCI) that may potentially be used for ecological monitoring of the populations of other threatened freshwater mussel species.

## Methods

### Data sampling

In this study, 3279 shells of *Margaritifera margaritifera* from 50 rivers and six countries across the freshwater pearl mussel range in Europe were used (Fig. 1A; Supplementary Tables 1 and 2; Dataset 1). A total of 2985 shells from 49 recent populations were studied (1984–2013; *n* > 10 specimens in each sample^34^). Additionally, we used 294 shells from 13 historical samples (~1840–1940; *n* ≥ 5 specimens in each sample^34^; each sample was collected from a certain site at the same time and represents a single population) found in the museum collections. We assessed shell lots from various museum collections as well as field samples of live mussels and shells. The living mussels were measured and returned to their habitat. The museum collections used are as follows:

IPEE - A.N. Severtsov Institute of Problems of Ecology and Evolution of the Russian Academy of Sciences, Moscow, Russia

MNHN – Muséum National d’Histoire Naturelle, Paris, France

NHMG – Göteborg Natural History Museum, Göteborg, Sweden

RMBH – Russian Museum of Biodiversity Hotspots, Federal Center for Integrated Arctic Research of the Russian Academy of Sciences, Arkhangelsk, Russia

SMNH – Swedish Museum of Natural History, Stockholm, Sweden

ULMHST – Museum of History of Science and Technology, University of Latvia, Riga, Latvia

ZIN – Zoological Institute of the Russian Academy of Sciences, St. Petersburg, Russia

We measured three shell dimensions for each specimen using calipers (±0.1 mm): the length (L), height (H), and width (W) of the shell, all taken at the maximal diameter^12,27^. To minimize morphological differences between populations resulting from the ontogenetic heterogeneity of their individuals^29,35^, all mussels with shell lengths less than 5 cm were excluded from the compiled data set.

### Morphometric analyses, population status assessment and general linear models (GLMs)

Using primary shell measurements, we calculated the basic morphometric index, namely the shell convexity index, SCI (W:L ratio × 100). In addition, we computed the integrated shell convexity index, SCI_I_:

$${{\rm{SCI}}}_{{\rm{I}}}={\rm{W}}/{{\rm{SA}}}^{1/2}\times 100,$$

where SA is a sagittal area calculated using an ellipse formula (π × L × H)^36^.

However, the SCI_I_ was found to be a function of the traditional SCI (Supplementary Fig. 1; Pearson’s *r* = 0.96, *n* = 62, *P* < 0.001) and was not used as a separate parameter. Additionally, we checked the assumption regarding the influence of sample size (number of mussels measured) on the index values using Spearman’s correlation test. We determined that the variability of both the mean SCI and SCI_I_ values are not correlated with sample size (Spearman’s *r* = 0.17, *n* = 62, *P* > 0.05).

Using the approach of Geist^6^, the status of recent populations was estimated as follows: (i) viable (functional) populations with high mussel density, and high or moderate recruitment, and (ii) declining (non-functional) populations with moderate or low mussel density, and low or no recruitment (see Supplementary Table 2 for details). We then calculated the mean shell index values for each population’s group separately. A Student’s *t*-test was used to check for differences between the means because the Kolmogorov-Smirnov and Lilliefors tests both revealed a normal distribution of morphometric indices at the intra-population and inter-population levels^34^ (STATISTICA 10, Stat Soft Inc., USA).

To estimate the relationship between the growth constant (K) and mean summer temperature, shell samples from 9 rivers (Keret’, Kozha, Maloshuika, Mutkajoki, Nimen’ga, Peypia, Solza, Somba, and Yud’ma) were investigated. Five specimens were used from each sample (*n* = 45 in total). For all samples, at least 16 annual rings for each shell were measured. The growth constants were calculated using a data approximation based on the recursive form of the Bertalanffy equation, i.e., the Ford-Walford equation^13^. The maximum age of individuals in a sample was calculated using a shell with the maximum length. Additionally, we incorporated a sclera-chronological approach by counting the annual rings in a thin transverse section of a valve^6,31^. In some cases, a logarithmic equation describing the trend line on the annual growth length vs. age plot was also applied.

To test the hypothesis of climate-induced morphometric differences between *M. margaritifera* populations the general linear models (GLMs; STATISTICA 10, Stat Soft Inc., USA) were used. We used shell width plotted against shell length as a covariate and population status as a factor with two levels (see Table 1 and Supplementary Table 2 for details) based on an approach described by Zieritz and Aldridge^36^. Additionally, we estimated differences between recent and historical samples using shell width plotted against shell length as a covariate and period of shell collecting as a factor with two levels. All GLMs were simplified to the minimal adequate models using sequential exclusion of insignificant factors from the model^37^. To test the assumption that latitude has different effects on the shell convexity at historical and recent time intervals, we applied a separate-slopes modeling approach (STATISTICA 10, Stat Soft Inc., USA) using the mean SCI as a dependent variable, latitude as a continuous predictor, and two-level time interval as a categorical predictor. Correlation of morphometric parameters with climatic and geographic variables was calculated using Pearson’s and Spearman’s coefficients depending on sample size, type of variables and normality test results.

### Modeling of freshwater pearl mussel response to climate change at the population level

Monthly mean air temperatures were obtained from the CRU TS v. 3.23 climate database (Climatic Research Unit, University of East Anglia) as gridded variables (0.5° resolution), which were based on weather station records during the period from 1 January 1901 to 31 December 2014^38^. For each location of the samples, we calculated the mean summer temperature (MST) values for the 10-, 20-, 30-, 40- and 50-year periods before the year of each sample collection (number of available samples varied depending on the period of averaging from 49 to 55) in accordance with the slow growth rate and large longevity of the freshwater pearl mussels^13,16,30^. These parameters were estimated as possible predictors for the mean SCI values in pearl mussel populations using a simple linear regression model algorithm of STATISTICA 10 (Stat Soft Inc., USA)^34^. The spatial auto-correlation in the modeling SCI data set (equation 1, see Results section) was accessed using Moran’s *I* index, which was calculated in ESRI ArcGIS 10. In addition to the MST, we tested some potentially important climatic parameters: mean temperature of spring, winter and autumn, annual mean temperature, monthly mean temperature, effective temperature sum (ETS) and thermal growing season length index (the two latter parameters were calculated for periods with temperatures above 5 °C and above 10 °C). The six samples from the River Kamp system (Austria) were excluded from the models in accordance with available recommendations^34^ because the three of them revealed outlier (anomalous) SCI values in relation to air temperature (Supplementary Fig. 2). To validate the resulting model inferred from the whole modeling data set (equation 1, see Results section), we used the k-fold cross-validation approach (k = 5)^39^. The standard prediction accuracy and error rate values of five training models were calculated using STATISTICA 10 (Stat Soft Inc., USA) and R language, i.e., mean absolute percentage deviation (MAPE), Tofallis’s relative accuracy measure, and min-max accuracy^40,41^.

### Modeling of spatial distribution of climatically suitable areas of freshwater pearl mussel populations at the continental scale

The general range model of *M. margaritifera* was processed using published mapping data^21^, which was transformed to a gridded digital map (0.5 × 0.5°) using ESRI ArcGIS 10 (Fig. 1A). Further, we simulated a spatial distribution of climatically suitable areas for *M. margaritifera* populations across Europe based on this digital range model, in each grid cell of which a mean SCI value was calculated using equation 1 and CRU TS v. 3.23′s MST data for each 20-year period, i.e., 1901–1920 and 1991–2010. The same modeling was performed for future climate scenarios (2061–2080) under three Representative Concentration Pathways (RCPs): low RCP 2.6, moderate RCP 4.5, and extreme RCP 8.5^42^. We used a set of gridded climate data (30 arcs) of the CMIP5 centennial simulations, which are based on the HadGEM2-ES global climate model, inferred from the WorldClim v. 1.4 database^43^.

Using the spatial models outlined above, climatically suitable areas were mapped based on the estimated threshold SCI values in viable and declining populations. These threshold values for the SCI interval in declining populations were as follows:

$${A}_{1}={A}_{2}+1.96\times {\rm{s}}{\rm{.e}}{\rm{.m}}. > {A}_{2}\ge {A}_{3}=({A}_{2}+{A}_{4})/2;$$

where *A*_1_ represents the estimated 95% upper confidence boundary of the mean SCI in declining populations which indicates disappeared populations under unsuitable thermal conditions for their existence; *A*_2_ represents the mean SCI in declining populations; *A*_3_ represents a threshold SCI value between viable and declining populations; *A*_4_ represents the mean SCI in viable populations; s.e.m. represents the standard error of the mean.

Accordingly, the estimated threshold value for the SCI interval in viable freshwater pearl mussel populations was as follows:

$${A}_{4} < {A}_{3}.$$

Here, we did not determine the lower limit of the mean SCI in viable populations because *M. margaritifera* is a cold-adapted species with the most abundant populations living in northern regions^13,14,16^.

Based on these intervals, we calculated the ranges of mean SCI values, i.e., SCI ≤28.37 and 28.38 ≤SCI ≤30.04 in viable and declining populations, respectively (*n* = 45, *P* = 0.05). The SCI values >30.04 indicate warm thermal conditions that likely unsuitable to populations’ survival at a 95% confidence level. These ranges were assigned for each grid cell located within the range model on a digital map.

## Electronic supplementary material

Supplementary Information

Supplementary Dataset 1
