# Supplementary material for: Climate Warming as a Possible Trigger of Keystone Mussel Population Decline in Oligotrophic Rivers at the Continental Scale
Source: Sci Rep. 2018 Jan 8;8:35. doi: 10.1038/s41598-017-18873-y (PMC5758527; doi:10.1038/s41598-017-18873-y)
Supplement: Supplementary file 1 — Supplementary Information [file 41598_2017_18873_MOESM1_ESM.pdf]

SUPPLEMENTARY INFORMATION

Climate Warming as a Possible Trigger of Keystone Mussel Population Decline in Oligotrophic Rivers at the Continental Scale

Ivan N. Bolotov,* Alexander A. Makhrov, Mikhail Yu. Gofarov, Olga V. Aksenova, Paul E. Aspholm, Yulia V. Bespalaya, Mikhail B. Kabakov, Yulia S. Kolosova, Alexander V. Kondakov, Thomas Ofenböck, Andrew N. Ostrovsky, Igor Yu. Popov, Ted von Proschwitz, Mudīte Rudzīte, Māris Rudzītis, Svetlana E. Sokolova, Ilmari Valovirta, Ilya V. Vikhrev, Maxim V. Vinarski and Alexey A. Zotin

*Corresponding author: inepras@yandex.ru

Contents

Supplementary Figure 1. Mean shell convexity index (SCI) vs. mean integrated shell convexity index (SCI_I) scatterplot in *Margaritifera margaritifera* populations from across Europe (full data set, *n* = 62).

Supplementary Figure 2. Scatterplot of mean summer temperature (MST_{20}, 20-year mean before sampling) vs. mean shell convexity index (SCI).

Supplementary Figure 3. Pearson's correlation coefficients of shell convexity index (SCI) and maximum age in recent *Margaritifera margaritifera* populations from across Europe with mean climatic variables for the 50-year period before sampling.

Supplementary Figure 4. Models of the mean shell convexity index (SCI) and maximum age of freshwater pearl mussels across Europe in the past using MST_{20} data obtained from CRU TS v. 3.23 climate database (Climatic Research Unit, University of East Anglia).

Supplementary Table 1. Summary of mean shell parameters, morphometric indices and maximum ages in *Margaritifera margaritifera* populations in the analyzed data set.

Supplementary Table 2. Assessment of the status of European *Margaritifera margaritifera* populations in the analyzed data set (recent samples).

Supplementary Table 3. Results of separate-slopes model (SSM) of the mean SCI in recent (1984-2013) and historical (~1840-1940) populations of *Margaritifera margaritifera*.

Supplementary Table 4. Results of k-fold cross-validation of equation 1 (k = 5) with parameters, significance, prediction accuracy and error rate values of five training models.

Supplementary Table 5. Pearson's correlation coefficients between the mean summer temperature (MST) during different periods before sample collection and the mean shell convexity index (SCI) and maximum age in *Margaritifera margaritifera* samples from across Europe ($p < 0.01$).

Supplementary Table 6. A review of temperature influence on the biological traits of *Margaritifera margaritifera*.

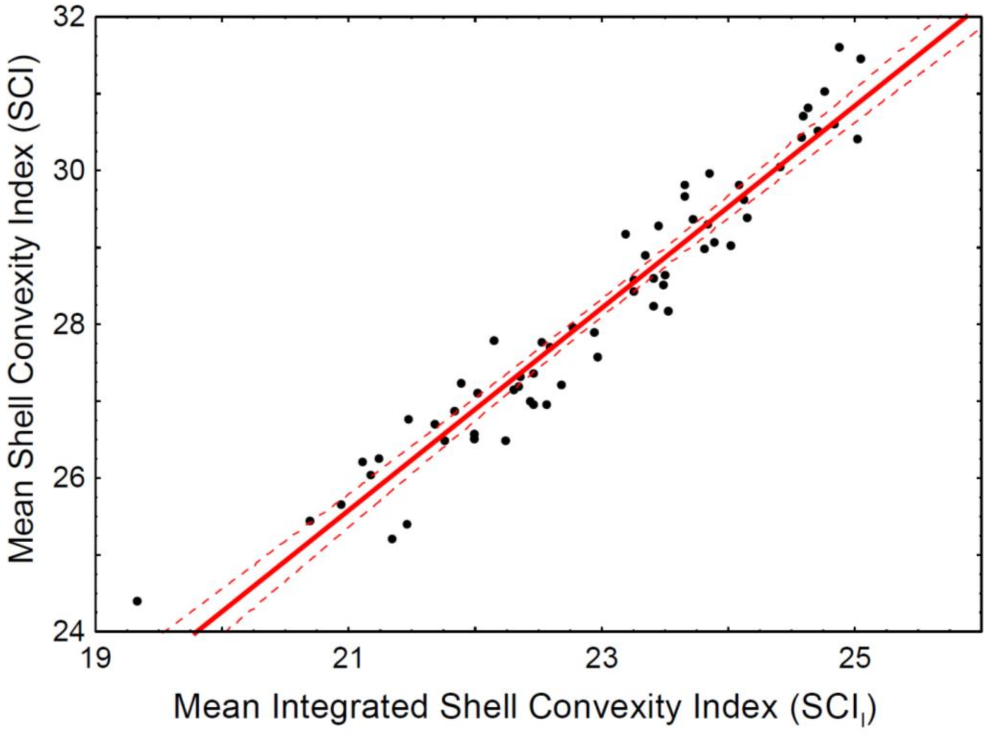

Supplementary Figure 1. Mean shell convexity index (SCI) vs. mean integrated shell convexity index (SCI$_I$) scatterplot in *Margaritifera margaritifera* populations from across Europe (full data set, *n* = 62). The dashed lines are the 95% confidence bounds of the regression model.

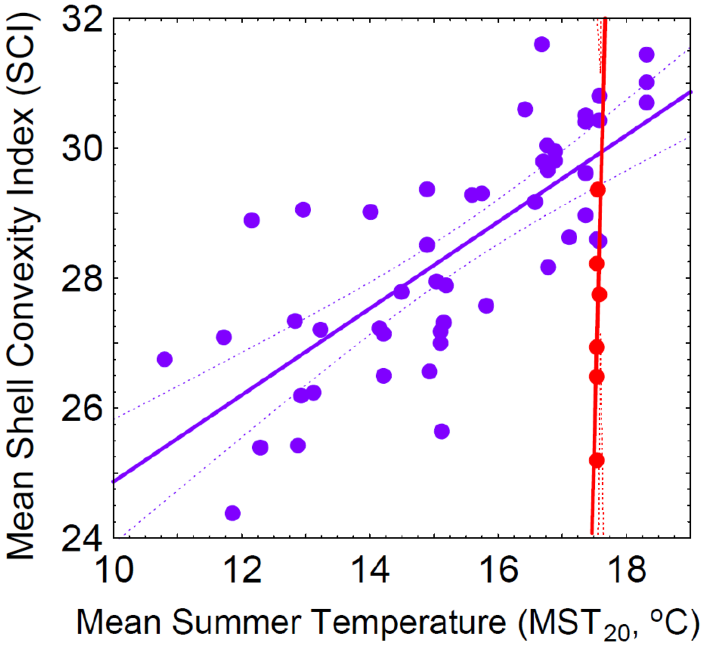

Supplementary Figure 2. Scatterplot of mean summer temperature (MST$_{20}$, 20-year mean before sampling) vs. mean shell convexity index (SCI). Each point represents the mean value in a population; red points are from the River Kamp system (*n* = 6), and violet points are from other European rivers (*n* = 49). The dashed lines are the 95% confidence bounds of the regression models.

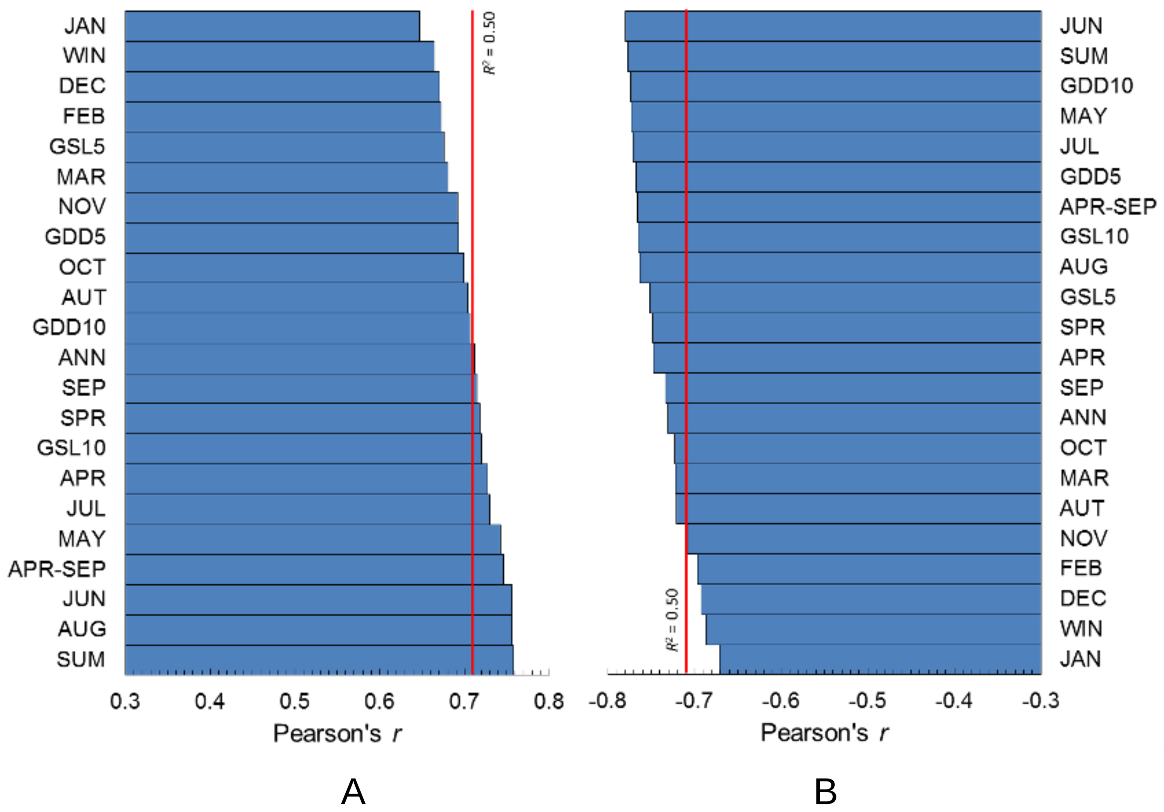

Supplementary Figure 3. Pearson's correlation coefficients of shell convexity index (SCI) and maximum age in recent *Margaritifera margaritifera* populations from across Europe with mean climatic variables for the 50-year period before sampling. (**A**) SCI (n = 43; $p < 0.001$). (**B**) Maximum age (n = 43; $p < 0.001$). Climatic parameters were averaged in accordance with the collecting year of each shell sample. Abbreviations: JAN … DEC – monthly mean air temperatures; WIN – mean winter temperature; SPR – mean spring temperature; SUM – mean summer temperature; AUT – mean autumn temperature; APR-SEP – mean temperature during growth season of mussels from April to September; GDD5 – effective temperature sum above 5°C; GSL5 – thermal growing season length index (above 5°C); GDD10 – effective temperature sum above 10°C; GSL10 – thermal growing season length index (above 10°C).

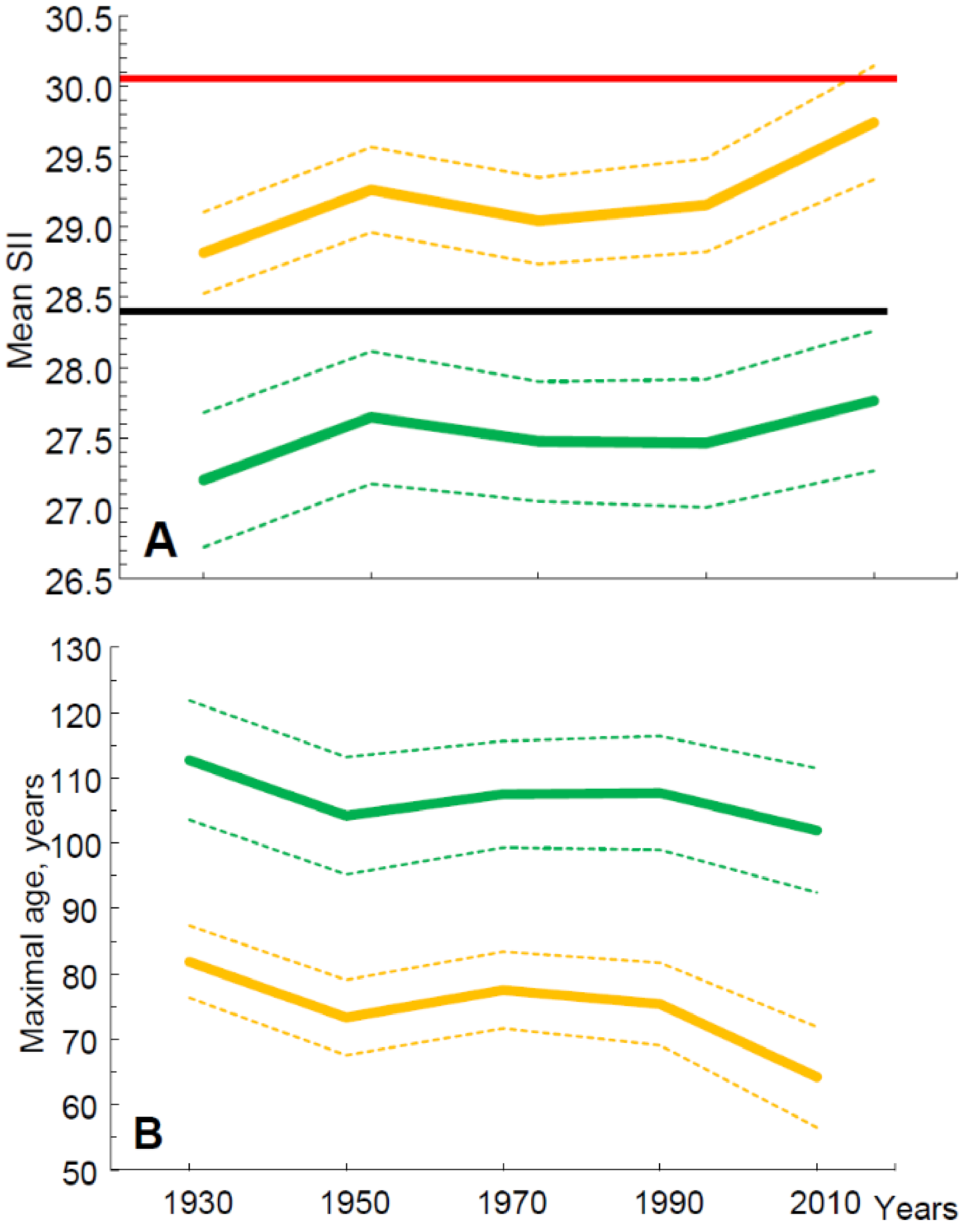

Supplementary Figure 4. Models of the mean shell convexity index (SCI) and maximum age of freshwater pearl mussels across Europe in the past using MST_{20} data obtained from CRU TS v. 3.23 climate database (Climatic Research Unit, University of East Anglia). (**A**) Long-term changes in the mean SCI. The mean index values were calculated using equation 1. The green line indicates recent viable populations (n = 15); the yellow line indicates recent declining populations (n = 24). The dashed lines are ±s.e.m. The red line indicates the 95% upper threshold SCI value corresponding to the expected upper limit of thermal stress on the population level. The black line indicates the mean SCI threshold value between viable and declining populations (n = 45). (**B**) Long-term changes in the maximum age. The age values were calculated using equation 2. The green line indicates recent viable populations (n = 15); the yellow line indicates recent declining populations (n = 24). The dashed lines are the 95% confidence intervals.

Supplementary Table 1. Summary of mean shell parameters, morphometric indices and maximum ages in *Margaritifera margaritifera* populations in the analyzed data set

Country	Drainage	River or stream	Locality	River site group*	Year of shell collecting	Period of shell collecting	Sample type**	Number of shells (N)	Coordinates (decimal degree)		Altitude, m a.s.l.	Shell length (L), mm				Max. shell height (H), mm				Shell width (W), mm				Shell convexity index (SCI = W/L×100)				Integrated shell convexity index ($SCI_I = W/SA^{1/2}×100$)				Max age, years	Source of max age assessment
									Latitude	Longitude		Mean	Min	Max	SD	Mean	Min	Max	SD	Mean	Min	Max	SD	Mean	Min	Max	SD	Mean	Min	Max	SD		
France	Atlantic Ocean	Loire River	Craponne-sur-Arzon	Upland	1873	Historical	MC (MNHN)	5	45.3319	3.8489	924	96.5	87.0	104.5	6.3	45.4	42.0	48.5	2.3	25.5	23.6	26.4	1.0	26.48	24.61	27.85	1.40	21.76	20.49	22.90	1.00	N/A	N/A
Latvia	Daugava River => Baltic Sea	Tumsupe Stream	Tumsupe 1 (old)	Lowland	1940	Historical	MC (ULMHST)	35	57.3551	26.0308	138	93.9	71.3	110.5	9.9	45.1	35.2	52.8	4.4	27.5	20.0	34.0	3.3	29.30	25.86	32.82	1.85	23.85	21.00	26.60	1.51	N/A	N/A
Russia	Baltic Sea	Sestra River	Near Hirole (old)	Lowland	1903	Historical	MC (ZIN)	8	60.1193	29.9571	7	98.2	79.2	117.2	11.4	47.2	39.1	53.2	4.4	26.1	21.9	29.7	2.4	26.69	25.05	28.28	1.08	21.68	20.94	22.63	0.60	N/A	N/A
Russia	Kola River => White Sea	Orlovka River	Orlovka (old)	Lowland	1926	Historical	MC (ZIN)	32	68.3854	33.3057	78	92.4	63.9	117.1	13.4	45.5	35.5	57.6	5.7	24.8	16.9	37.6	4.2	26.75	24.09	32.14	1.49	21.48	18.91	26.34	1.30	N/A	N/A
Russia	Solza River => White Sea	Kazanka River	Kazanka (old)	Lowland	1936	Historical	MC (ZIN)	11	64.2623	39.1059	93	107.7	84.3	121.8	11.7	53.1	42.4	63.7	6.6	29.4	22.9	36.6	4.1	27.23	24.17	30.18	1.71	21.89	19.70	23.82	1.27	N/A	N/A
Russia	Umba River => White Sea	Lyamuksa River	Lyamuksa (old)	Lowland	1936	Historical	MC (ZIN)	48	66.8685	34.5818	84	77.4	55.3	102.1	9.8	37.3	29.3	45.9	3.6	21.0	14.6	27.1	2.9	27.09	24.31	29.74	1.22	22.03	17.88	25.45	1.78	N/A	N/A
Russia	White Sea	Por'ja River	Por'ja Bay (old)	Lowland	1868	Historical	MC (ZIN)	11	66.8247	33.5395	7	118.6	79.3	134.3	14.8	57.1	40.3	63.7	6.7	32.0	20.8	37.6	4.9	26.87	24.10	29.03	1.50	21.84	19.71	23.90	1.19	N/A	N/A
Russia	White Sea	Zhemchuzhnaya River	Zhemchuzhnaya (old)	Lowland	1930	Historical	MC (ZIN)	14	66.8859	32.4654	11	75.9	51.2	118.5	21.4	38.0	27.1	53.6	8.9	18.7	11.9	32.3	6.1	24.38	22.45	27.28	1.43	19.33	17.68	23.17	1.37	163	Ref. 44
Sweden	Baltic Sea	Bräkneån Stream	West of Öljehult	Lowland	1880	Historical	MC (SMNH)	25	56.4165	15.0057	103	111.1	99.1	125.2	7.3	50.3	42.4	55.7	3.2	29.9	21.4	35.1	2.7	26.94	18.94	29.46	2.12	22.57	17.44	24.62	1.49	N/A	N/A
Sweden	Göta-älv River => North Sea	Bäckeforsån Stream	East of Bäcke church	Lowland	~1840	Historical	MC (SMNH)	25	58.7951	12.1664	150	102.9	85.2	115.2	10.5	48.8	41.3	54.7	4.3	29.3	23.3	34.3	3.7	28.42	25.95	35.34	1.75	23.26	21.23	28.20	1.36	N/A	N/A
Sweden	Sommen Lake => Motala Ström River => Baltic Sea	Bulsjöån Stream	East of Norra Vi Church	Lowland	~1840	Historical	MC (SMNH)	25	57.8835	15.3840	175	105.7	76.5	120.9	9.7	50.5	33.0	56.5	4.5	29.2	21.4	32.9	2.3	27.69	26.09	30.32	1.04	22.60	21.51	24.03	0.68	N/A	N/A
Sweden	Suseån River => North Sea	Vallebäcken Stream	Vallebäcken 1	Lowland	~1845	Historical	MC (NHMG)	30	56.8102	12.7876	53	87.9	65.2	117.1	12.5	42.1	31.6	53.8	5.2	22.9	13.7	32.1	4.0	26.02	21.01	29.67	1.94	21.18	17.03	24.11	1.53	N/A	N/A
Sweden	Viskan River => North Sea	Ringsbäcken Stream	Björnåsa	Lowland	1919	Historical	MC (SMNH)	25	57.6596	12.9816	160	80.1	52.7	107.2	14.1	38.1	26.9	51.6	6.0	20.7	13.2	30.9	4.5	25.64	23.15	28.82	1.43	20.94	18.77	23.48	1.25	N/A	N/A
Austria	Danube River => Black Sea	Aubach Stream	Downstream of Leithen	Upland	1992	Recent	FM	88	48.4393	13.6305	436	101.2	60.0	118.0	10.4	49.0	33.0	58.0	4.2	30.8	18.0	37.0	3.4	30.50	25.42	34.78	1.92	24.71	19.59	27.62	1.53	65	Original
Austria	Danube River => Black Sea	Daglesbach Stream	Downstream of Lembach	Upland	1992	Recent	FM	40	48.4930	13.8930	538	98.5	58.0	112.0	10.9	46.4	32.0	55.0	5.2	28.5	15.0	33.0	3.5	28.97	23.86	32.14	1.80	23.81	19.64	26.84	1.52	57	Original
Austria	Danube River => Black Sea	Feldaist Stream	Upstream of Rainbach	Upland	1992	Recent	FM	63	48.5576	14.4872	667	96.5	69.0	112.0	6.0	46.6	34.0	55.0	3.0	29.5	21.0	35.0	2.1	30.60	28.00	33.65	1.17	24.85	22.80	27.29	0.95	64	Original
Austria	Danube River => Black Sea	Großer Kamp Stream	Neustift/Hausbach	Upland	1992	Recent	FM	47	48.5087	15.0381	699	95.1	58.0	113.0	12.6	43.8	29.0	52.0	5.0	26.8	17.0	32.0	3.8	28.22	22.99	31.40	1.61	23.42	18.89	26.61	1.37	54	Original
Austria	Danube River => Black Sea	Harbe Aist River	Neuhof	Upland	1992	Recent	FM	33	48.5209	14.6684	876	100.5	82.0	112.0	8.8	50.3	42.0	58.0	3.9	30.0	26.0	35.0	2.3	29.95	24.07	32.97	1.92	23.86	20.81	25.98	1.26	42	Original
Austria	Danube River => Black Sea	Kamp River	Utissenbach	Upland	1992	Recent	FM	51	48.5623	15.1179	591	97.0	55.0	113.0	13.4	43.5	27.0	51.0	5.0	25.6	16.0	32.0	3.3	26.48	23.08	30.00	1.45	22.25	19.58	24.56	1.00	46	Original
Austria	Danube River => Black Sea	Kleine Naarn Stream	Upstream of Steinbruckmühle	Upland	1992	Recent	FM	22	48.3469	14.7192	489	68.0	54.0	100.0	11.5	33.1	26.0	49.0	5.3	20.7	16.0	30.0	3.5	30.43	27.12	32.61	1.56	24.59	22.06	27.23	1.32	45	Original
Austria	Danube River => Black Sea	Kleine Rodl Stream	Upstream of Eidendorf	Upland	1992	Recent	FM	48	48.4278	14.1391	525	95.9	68.0	110.0	8.7	48.1	35.0	57.0	4.4	30.1	21.0	35.0	3.0	31.44	27.27	35.56	1.84	25.05	22.80	28.83	1.45	57	Original
Austria	Danube River => Black Sea	Pesenbach Stream	Gerling	Upland	1992	Recent	FM	30	48.4029	14.0814	463	79.2	55.0	95.0	7.2	39.3	28.0	46.0	3.8	24.3	17.0	30.0	2.3	30.70	28.40	34.72	1.33	24.60	22.25	27.19	1.09	40	Original
Austria	Danube River => Black Sea	Pesenbach Stream	Waldhofer	Upland	1992	Recent	FM	25	48.4346	14.0807	506	85.3	59.0	122.0	16.1	42.4	32.0	57.0	6.7	26.4	19.0	39.0	5.1	31.01	28.21	34.29	1.43	24.76	21.95	26.97	1.17	57	Original
Austria	Danube River => Black Sea	Pfudabach Stream	Upstream of Angsüß	Upland	1992	Recent	FM	36	48.4102	13.6238	346	105.3	85.0	126.0	8.4	49.4	43.0	58.0	3.7	31.9	25.0	38.0	2.9	30.40	25.00	36.47	2.35	25.02	21.03	28.60	1.66	65	Original
Austria	Danube River => Black Sea	Pfudabach Stream	Upstream of Mitterndorf	Upland	1992	Recent	FM	35	48.4203	13.6254	356	104.4	83.0	121.0	9.0	49.9	40.0	57.0	3.9	30.9	23.0	35.0	3.2	29.62	23.85	35.35	2.55	24.13	20.45	27.79	1.75	60	Original
Austria	Danube River => Black Sea	Purzelkamp Stream	Kl. Weißenbach	Upland	1992	Recent	FM	40	48.5109	15.2193	708	89.4	80.0	97.0	4.8	39.6	36.0	43.0	1.8	22.5	19.0	26.0	1.6	25.20	21.65	31.33	1.62	21.35	18.79	26.84	1.34	41	Original
Austria	Danube River => Black Sea	Purzelkamp Stream	Rappoltschlag	Upland	1992	Recent	FM	33	48.5154	15.2525	696	89.9	77.0	108.0	7.4	42.6	34.0	51.0	3.9	25.7	18.0	32.0	3.3	28.60	22.99	35.23	2.72	23.41	19.72	26.91	1.72	42	Original
Austria	Danube River => Black Sea	Purzelkamp Stream	Grafenschlag	Upland	1992	Recent	FM	27	48.4906	15.1787	742	82.9	58.0	98.0	9.3	40.3	30.0	48.0	4.1	24.3	18.0	29.0	2.7	29.36	27.06	31.08	1.10	23.72	21.98	25.50	0.83	46	Original
Austria	Danube River => Black Sea	Purzelkamp Stream	Watermill near Rappoltschlag	Upland	1992	Recent	FM	55	48.5197	15.2484	682	92.6	79.0	109.0	7.0	42.1	33.0	51.0	4.4	25.0	17.0	33.0	4.3	26.94	21.25	32.50	3.94	22.47	18.39	25.93	2.50	43	Original
Austria	Danube River => Black Sea	Waldaist River	Feiblmühl	Upland	1992	Recent	FM	52	48.3912	14.6352	437	66.4	51.0	116.0	11.6	32.9	26.0	55.0	5.1	20.2	16.0	28.0	2.7	30.80	24.14	40.68	3.65	24.64	19.55	31.66	2.68	50	Original
Austria	Danube River => Black Sea	Waldaist River	Haidmühle	Upland	1992	Recent	FM	61	48.4309	14.7127	592	80.0	51.0	122.0	12.7	38.2	27.0	53.0	5.2	22.7	17.0	36.0	3.3	28.57	21.52	40.00	2.70	23.27	18.24	31.40	2.01	60	Original
Austria	Danube River => Black Sea	Waldaist River	Riedelhammer	Upland	1992	Recent	FM	58	48.4200	14.6386	525	72.4	51.0	113.0	13.7	34.9	27.0	55.0	6.3	20.0	14.0	30.0	3.6	27.75	23.81	36.51	1.62	22.53	19.47	28.46	1.16	58	Original
Austria	Elbe River => North Sea	Lainsitz Stream	St. Martin	Upland	1992	Recent	FM	35	48.6641	14.8381	615	91.4	71.0	113.0	10.6	46.2	37.0	56.0	5.1	27.2	22.0	34.0	3.1	29.81	26.67	32.98	1.53	23.66	21.04	26.60	1.35	51	Original
Finland	Iijoki River => Baltic Sea	Livojoki Stream	Livojoki 1	Upland	1989	Recent	FM	32	65.9003	27.5018	202	100.3	65.5	117.0	9.8	47.1	32.0	54.0	4.1	29.1	20.0	35.0	3.2	29.05	26.00	33.02	1.53	23.90	20.74	27.26	1.26	N/A	N/A
Finland	Kem' River => White Sea	Juomajoki Stream	Juomajoki 1	Upland	1984	Recent	FM	30	66.0140	28.9292	311	87.0	70.0	126.5	13.7	42.2	34.5	58.0	5.5	25.0	20.0	35.5	3.3	28.89	26.03	32.14	1.43	23.35	21.13	26.28	1.01	N/A	N/A
France	Loire River => Atlantic Ocean	Dolore River	Dolore	Upland	1994	Recent	MC (MNHN)	11	45.4513	3.7327	548	79.0	61.1	92.1	9.6	39.3	32.9	45.0	3.7	23.3	20.1	26.1	2.2	29.66	25.15	34.10	2.43	23.67	20.76	27.10	1.56	N/A	N/A
Latvia	Daugava River => Baltic Sea	Pededze Stream	Pededze 1	Lowland	2012	Recent	FM	15	56.9372	26.8992	87	110.4	83.5	129.5	10.5	52.0	40.5	60.5	4.3	31.6	24.0	38.5	3.4	28.63	26.01	30.80	1.31	23.51	21.91	25.31	1.01	N/A	N/A
Latvia	Daugava River => Baltic Sea	Tumsupe Stream	Tumsupe 1	Lowland	2011	Recent	FM	42	57.3551	26.0308	138	88.8	67.5	105.0	8.6	42.7	35.0	49.0	3.4	26.7	20.5	32.0	2.9	30.04	25.60	36.60	2.18	24.42	20.42	29.90	1.76	N/A	N/A
Latvia	Gauja River => Baltic Sea	Perlupe Stream	Perlupe 1	Lowland	2010	Recent	FM	55	57.1950	25.2201	132	101.4	83.0	121.5	9.1	52.0	41.0	61.0	4.0	32.0	25.0	41.5	3.3	31.60	27.75	38.55	2.21	24.88	21.35	30.95	1.67	N/A	N/A
Latvia	Gauja River => Baltic Sea	Rauza River	Rauza 1-2	Lowland	2009-2012	Recent	FM	147	57.0051	24.5726	26	99.3	61.0	126.0	13.6	48.3	29.0	61.5	6.3	29.6	17.0	39.0	4.5	29.80	22.60	36.81	2.04	24.09	18.73	28.46	1.48	N/A	N/A
Russia	Baltic Sea	Peypia River	Peypia 1	Lowland	2011	Recent	MC (RMBH)	112	59.7694	28.7205	4	106.5	76.0	130.8	12.3	48.6	34.9	62.8	5.6	30.1	20.1	40.7	4.5	28.17	23.28	33.05	2.00	23.54	19.05	27.99	1.74	95	Original
Russia	Neva River => Baltic Sea	Khorinka River	Khorinka 1	Lowland	2011-2012	Recent	MC (IPEE)	78	58.4530	33.3627	92	75.0	55.4	106.1	12.6	37.7	27.5	51.6	6.0	22.0	14.0	33.9	4.5	29.17	24.20	34.60	2.15	23.20	19.58	27.62	1.85	76	Original
Russia	Neva River => Baltic Sea	Shotkusa River	Shotkusa 1	Lowland	2011-2012	Recent	MC (IPEE)	30	60.5572	33.2583	17	122.6	76.8	135.7	11.7	60.7	41.3	67.6	5.2	35.8	23.9	42.0	3.1	29.28	26.58	32.60	1.78	23.45	21.51	25.69	1.20	76	Original
Russia	Nimen'ga River => White Sea	Yud'ma Stream	Yud'ma 1	Lowland	2011	Recent	FM	178	63.6627	37.6328	40	100.2	69.0	134.2	11.3	47.1	34.7	61.6	4.7	27.2	18.4	35.0	3.2	27.18	24.08	31.60	1.38	22.36	20.17	25.84	1.11	105	Original
Russia	Onega River => White Sea	Kozha River	Kozha 3	Lowland	2010	Recent	FM	45	63.2392	38.0683	88	98.9	52.6	112.7	11.0	46.5	26.2	55.4	5.0	29.1	14.4	33.5	3.4	29.37	26.84	31.98	1.17	24.15	21.56	26.51	1.02	74	Original
Russia	Onega River => White Sea	Kozha River	Kozha 7	Lowland	2010	Recent	FM	51	63.3525	38.2214	77	95.3	71.5	120.8	11.7	44.6	34.1	55.5	5.1	27.1	20.8	33.8	3.3	28.51	23.51	33.98	2.18	23.49	19.05	27.75	1.67	74	Original
Russia	Onega River => White Sea	Kozha River	Kozha 9	Lowland	2010	Recent	FM	37	63.5936	38.3825	30	85.7	71.2	109.9	10.0	41.0	31.6	49.8	4.1	23.9	18.6	29.6	2.9	27.95	24.90	32.55	1.79	22.78	20.21	26.00	1.51	74	Original
Russia	Onega River => White Sea	Somba River	Somba	Lowland	2013	Recent	FM	20	62.9437	39.5138	13	113.1	90.6	129.6	9.4	53.7	44.2	61.0	3.7	30.9	21.0	37.2	3.6	27.32	18.68	30.46	2.52	22.37	15.18	25.15	2.11	93	Original
Russia	Paanajarvi Lake => White Sea	Mutkajoki River	Mutkajoki 1	Lowland	2011	Recent	FM	111	66.2845	30.3451	154	111.2	57.1	136.0	19.0	52.3	29.2	65.0	8.3	30.5	16.1	39.2	5.7	27.34	23.38	32.61	1.54	22.47	19.39	26.67	1.29	179	Original
Russia	Solza River => White Sea	Kazanka River	Kazanka 1	Lowland	2006	Recent	FM	138	64.2536	39.1487	134	98.8	76.0	136.3	12.9	45.5	34.0	60.5	5.2	26.2	20.0	37.1	4.0	26.50	22.08	32.00	1.74	22.00	19.10	25.43	1.38	N/A	N/A
Russia	Solza River => White Sea	Kazanka River	Kazanka 2	Lowland	2006	Recent	FM	59	64.2623	39.1059	93	90.7	57.4	128.0	19.2	42.5	23.0	57.0	8.1	24.7	15.0	36.0	6.1	27.14	22.60	31.41	1.87	22.31	19.00	25.88	1.57	N/A	N/A
Russia	White Sea	Gridina River	Gridina 1	Lowland	2007	Recent	MC (IPEE)	45	65.9101	34.6217	18	110.7	96.1	122.6	6.4	50.7	44.7	57.2	2.7	30.1	25.0	34.8	2.4	27.21	23.87	31.98	1.66	22.69	20.08	26.36	1.37	N/A	N/A
Russia	White Sea	Keret' River	Keret' 1	Lowland	2005	Recent	MC (IPEE)	22	66.2747	33.5622	18	89.2	52.0	115.0	16.7	42.9	26.0	57.2	8.0	22.8	13.0	34.5	5.1	25.42	22.03	30.00	1.78	20.70	17.86	24.70	1.61	114	Ref. 45
Russia	White Sea	Keret' River	Keret' 2	Lowland	2006	Recent	MC (IPEE)	18	66.2746	33.5615	18	86.4	51.0	116.0	14.7	42.2	26.0	54.0	6.5	22.6	13.0	28.0	3.7	26.19	24.14	28.57	1.08	21.11	19.96	22.95	0.84	114	Ref. 45
Russia	White Sea	Maloshuika River	Maloshuika 1	Lowland	2011	Recent	FM	224	63.6816	37.4061	45	113.3	52.7	142.9	12.0	52.5	26.7	65.9	5.3	30.1	13.2	39.4	3.8	26.56	21.28	31.46	1.59	22.00	17.91	25.83	1.26	199	Original
Russia	White Sea	Nimen'ga River	Nimen'ga 1	Lowland	2011	Recent	FM	277	63.6646	37.6289	40	113.0	79.9	135.0	10.2	52.0	37.9	62.5	4.3	30.5	20.5	38.2	3.1	27.00	22.12	31.84	1.64	22.44	18.24	26.61	1.28	121	Original
Russia	White Sea	Solza River	Solza 1	Lowland	2011	Recent	FM	91	64.4774	39.5440	14	86.9	51.4	114.5	16.2	43.3	26.2	56.8	7.4	24.1	15.1	33.7	4.5	27.79	21.27	32.49	1.73	22.16	17.98	25.10	1.20	69	Original
Russia	White Sea	Varzuga River	Varzuga 1	Lowland	2012	Recent	FM	58	66.8869	35.9287	74	76.0	55.0	108.5	14.4	33.7	25.7	45.9	5.5	19.3	13.8	27.2	3.8	25.39	23.26	29.11	1.36	21.47	19.33	24.39	1.21	190	Ref. 45
Sweden	Baltic Sea	Trönöån River	Trönöån 1	Lowland	2010	Recent	MC (NHMG)	30	61.4613	16.7022	167	122.7	96.7	138.0	10.7	57.0	45.8	67.5	4.7	35.7	23.5	46.5	4.9	29.02	24.30	33.70	1.93	24.02	19.92	27.87	1.66	N/A	N/A
Sweden	Dalälven River => Baltic Sea	Hyttkvarnsån Stream	Hyttkvarnsån 1	Upland	1996	Recent	MC (NHMG)	30	60.4456	15.1541	350	89.7	71.9	101.6	8.9	43.5	36.1	49.4	3.7	23.5	17.6	28.7	2.7	26.24	23.72	34.70	2.12	21.25	19.19	27.38	1.64	N/A	N/A
Sweden	Viskan River => North Sea	Iglabäcken Stream	Iglabäcken 1	Lowland	2013	Recent	FM	90	57.5167	12.5447	45	91.9	61.6	110.1	8.1	42.1	31.7	49.9	3.7	25.3	18.2	29.3	2.4	27.57	24.83	33.53	1.46	22.97	20.31	27.91	1.16	N/A	N/A
Sweden	Viskan River => North Sea	Mäsån Stream	Mäsån 1	Lowland	2008	Recent	MC (NHMG)	30	59.0605	12.3541	181	93.1	81.5	105.9	5.2	43.8	38.2	49.6	2.3	26.0	21.9	30.4	1.8	27.89	24.12	31.29	1.62	22.95	19.80	25.78	1.25	N/A	N/A

*River site group: upland is >200 m alt.; lowland is <200 m alt. **Sample type: FM - field measurements; MC - museum collection (museum abbreviations are given in parentheses). N/A - not available.

Supplementary Table 2. Assessment of the status of European *Margaritifera margaritifera* populations in the analyzed data set (recent samples)

Country	Drainage	River or stream	Locality	River site group*	Year of shell collecting	Population status**	Time period of the population status assessment	References
Austria	Danube River => Black Sea	Aubach Stream	Downstream of Leithen	Upland	1992	declining	1992-2002	Refs. 26, 46
Austria	Danube River => Black Sea	Daglesbach Stream	Downstream of Lembach	Upland	1992	declining	1992-2002	Refs. 46, 47
Austria	Danube River => Black Sea	Feldaist Stream	Upstream of Rainbach	Upland	1992	viable	1992-2002	Refs. 26, 46
Austria	Danube River => Black Sea	Großer Kamp Stream	Neustift/Hausbach	Upland	1992	viable	1992-2002	Refs. 26, 46, 47
Austria	Danube River => Black Sea	Harbe Aist River	Neuhof	Upland	1992	declining	1992-2002	Refs. 26, 46, 47
Austria	Danube River => Black Sea	Kamp River	Utissenbach	Upland	1992	viable	1992-2002	Refs. 26, 46, 47
Austria	Danube River => Black Sea	Kleine Naarn Stream	Upstream of Steinbruckmühle	Upland	1992	declining	1992-2002	Refs. 26, 46
Austria	Danube River => Black Sea	Kleine Rodl Stream	Upstream of Eidendorf	Upland	1992	declining	1992-2002	Refs. 26, 46
Austria	Danube River => Black Sea	Pesenbach Stream	Gerling	Upland	1992	declining	1992-2002	Refs. 26, 46
Austria	Danube River => Black Sea	Pesenbach Stream	Waldhofer	Upland	1992	declining	1992-2002	Refs. 26, 46
Austria	Danube River => Black Sea	Pfudabach Stream	Upstream of Angsüß	Upland	1992	declining	1992-2002	Refs. 26, 46
Austria	Danube River => Black Sea	Pfudabach Stream	Upstream of Mitterndorf	Upland	1992	declining	1992-2002	Refs. 26, 46
Austria	Danube River => Black Sea	Purzelkamp Stream	Kl. Weißenbach	Upland	1992	viable	1992-2002	Refs. 26, 46
Austria	Danube River => Black Sea	Purzelkamp Stream	Rappoltschlag	Upland	1992	viable	1992-2002	Refs. 26, 46
Austria	Danube River => Black Sea	Purzelkamp Stream	Grafenschlag	Upland	1992	viable	1992-2002	Refs. 26, 46
Austria	Danube River => Black Sea	Purzelkamp Stream	Watermill near Rappoltschlag	Upland	1992	viable	1992-2002	Refs. 26, 46
Austria	Danube River => Black Sea	Waldaist River	Feiblmühl	Upland	1992	declining	1992-2002	Refs. 26, 46-49
Austria	Danube River => Black Sea	Waldaist River	Haidmühle	Upland	1992	declining	1992-2007	Refs. 26, 46-49
Austria	Danube River => Black Sea	Waldaist River	Riedelhammer	Upland	1992	declining	1992-2007	Refs. 26, 46-49
Austria	Elbe River => North Sea	Lainsitz Stream	St. Martin	Upland	1992	declining	1992-2002	Refs. 26, 46, 47
Finland	Iijoki River => Baltic Sea	Livojoki Stream	Livojoki 1	Upland	1989	N/A	N/A	N/A
Finland	Kem' River => White Sea	Juomajoki Stream	Juomajoki 1	Upland	1984	N/A	N/A	N/A
France	Loire River => Atlantic Ocean	Dolore River	Dolore	Upland	1994	N/A	N/A	N/A
Latvia	Daugava River => Baltic Sea	Pededze Stream	Pededze 1	Lowland	2012	declining	1999-2012	Refs. 50, 51
Latvia	Daugava River => Baltic Sea	Tumsupe Stream	Tumsupe 1	Lowland	2011	declining	1999-2011	Refs. 51, 53
Latvia	Gauja River => Baltic Sea	Perlupe Stream	Perlupe 1	Lowland	2010	declining	1977-2010	Refs. 50-52
Latvia	Gauja River => Baltic Sea	Rauza River	Rauza 1-2	Lowland	2009-2012	declining	1999-2012	Refs. 50, 52
Russia	Baltic Sea	Peypia River	Peypia 1	Lowland	2011	viable	2008-2009	Refs. 53, 54
Russia	Neva River => Baltic Sea	Khorinka River	Khorinka 1	Lowland	2011-2012	declining	2011-2012	Ref. 54
Russia	Neva River => Baltic Sea	Shotkusa River	Shotkusa 1	Lowland	2011-2012	declining	2009-2012	Ref. 54
Russia	Nimen'ga River => White Sea	Yud'ma Stream	Yud'ma 1	Lowland	2011	viable	2011	Ref. 14
Russia	Onega River => White Sea	Kozha River	Kozha 3	Lowland	2010	declining	2009-2010	Refs. 14, 55
Russia	Onega River => White Sea	Kozha River	Kozha 7	Lowland	2010	declining	2009-2010	Refs. 14, 55
Russia	Onega River => White Sea	Kozha River	Kozha 9	Lowland	2010	declining	2009-2010	Refs. 14, 55
Russia	Onega River => White Sea	Somba River	Somba	Lowland	2013	declining	2013	Unpubl. data
Russia	Paanajarvi Lake => White Sea	Mutkajoki River	Mutkajoki 1	Lowland	2011	viable	2011	Ref. 14
Russia	Solza River => White Sea	Kazanka River	Kazanka 1	Lowland	2006	viable	1998-2006	Refs. 14, 59
Russia	Solza River => White Sea	Kazanka River	Kazanka 2	Lowland	2006	viable	1998-2006	Refs. 14, 59
Russia	White Sea	Gridina River	Gridina 1	Lowland	2007	viable	2007	Ref. 14
Russia	White Sea	Keret' River	Keret' 1	Lowland	2005	viable	1990-2006	Refs. 13, 14
Russia	White Sea	Keret' River	Keret' 2	Lowland	2006	viable	1990-2006	Refs. 13, 14
Russia	White Sea	Maloshuika River	Maloshuika 1	Lowland	2011	viable	2011	Ref. 14
Russia	White Sea	Nimen'ga River	Nimen'ga 1	Lowland	2011	viable	2011	Ref. 14
Russia	White Sea	Solza River	Solza 1	Lowland	2011	viable	2005-2011	Refs. 14, 59
Russia	White Sea	Varzuga River	Varzuga 1	Lowland	2012	viable	1989-2012	Refs. 13, 14
Sweden	Baltic Sea	Trönöån River	Trönöån 1	Lowland	2010	N/A	N/A	N/A
Sweden	Dalälven River => Baltic Sea	Hyttkvarnsån Stream	Hyttkvarnsån 1	Upland	1996	viable***	1996	Ref. 56
Sweden	Viskan River => North Sea	Iglabäcken Stream	Iglabäcken 1	Lowland	2013	viable	2006-2013	Ref. 57
Sweden	Viskan River => North Sea	Mäsån Stream	Mäsån 1	Lowland	2008	declining	2008	Ref. 58

*River site group: upland is >200 m alt.; lowland is <200 m alt. Population status (assessment to the date of the shell collection): **viable - high density, high or moderate recruitment; declining - moderate or low density, low or no recruitment; and ***low recruitment because of very low density of native host stock in the stream due to overfishing, but the artificial recruitment was successful, which reveals suitable conditions for the mussel reproduction. N/A - not available.

Supplementary Table 3. Results of separate-slopes model of the mean SCI in recent (1984-2013) and historical (~1840-1940) populations of *Margaritifera margaritifera*.

Response variable	Source	*SS*	*d.f.*	*F*	*P*
Mean SCI	Intercept	218.2	1	192.1	<0.001
	Sample Group* × Latitude	32.1	2	14.1	<0.001
	Latitude	9.2	1	8.1	0.007
	Error	37.5	33.0		

*Historical samples (lowland rivers) vs. recent samples (lowland rivers) as a two-level categorical predictor.

Supplementary Table 4. Results of k-fold cross-validation of equation 1 (k = 5) with parameters, significance, prediction accuracy and error rate values of five training models.

**Training model**	**Intercept α**	**Slope β**	**Pearson *r***	***F***	***P***	**Mean absolute percentage deviation (MAPE), %**	**Min – max accuracy**	**Tofallis's relative accuracy measure (Σ(ln Q)^2)**
No. 1	16.691	0.757	0.83	83.9	<0.001	5.49	0.95	0.04
No. 2	19.566	0.585	0.70	36.0	<0.001	4.02	0.97	0.01
No. 3	18.722	0.635	0.75	47.2	<0.001	3.55	0.97	0.01
No. 4	18.656	0.636	0.72	41.5	<0.001	2.67	0.97	0.01
No. 5	17.168	0.734	0.79	63.3	<0.001	2.28	0.96	0.02

Supplementary Table 5. Pearson's correlation coefficients between the mean summer temperature (MST) during different periods before sample collection and the mean shell convexity index (SCI) and maximum age in *Margaritifera margaritifera* samples from across Europe ($p < 0.01$). *N* is number of samples.

**Parameters**	**Mean SCI**	**Max age**	***N***
MST_{10} (10-year mean before sampling)	0.731	-0.767	49
MST_{20} (20-year mean before sampling)	0.756	-0.769	49
MST_{30} (30-year mean before sampling)	0.761	-0.775	47
MST_{40} (40-year mean before sampling)	0.762	-0.778	44
MST_{50} (50-year mean before sampling)	0.767	-0.783	43

Supplementary Table 6. A review of temperature influence on the biological traits of *Margaritifera margaritifera*.

Biological trait	Region	Response to temperature influence	References
Longevity	Europe	Longevity in southern rivers is strongly reduced in comparison with northern rivers	Ref. 30
Longevity	Spain	Southernmost populations exhibit the highest growth rates, together with the lowest maximum age and maximum length	Ref. 60
Metabolic rate	Europe	Metabolic rate reveals positive correlation with temperature	Ref. 30
Growth period	Germany	Growth is restricted to the warm period of the year and decreased to almost zero from October to March	Ref. 24
Growth period	Germany	Growth in warmer periods from May/June to October/November is greater than from October/November to May/June	Ref. 61
Growth rate	Sweden	Growth is influenced by temperature with faster rates of shell growth during warmer summers. Standardized annual growth rates and air temperature, which covaries with water temperature, exhibit a significant positive correlation	Ref. 6
Growth rate	Finland	Annual increments are significantly narrower in the north (Finland) than in the south (Spain). The oldest, largest and slowest growing populations can be found in the proximity of the northernmost distribution limit of the species.	Ref. 31
Growth rate	Germany	Growth constant reveals a positive correlation with temperature; slower mussel growth found in the upper parts of rivers and increased growth downstream	Refs. 24, 30, 62–63
Spawning (oviposition)	Scotland	The timing of spawning is determined gradually, probably by a thermal summation effect	Ref. 64
Glochidial release (spat)	Scotland	Mussels in the warmest rivers tend to release glochidia earlier than those inhabiting colder rivers. Thermal variations also seem to influence the timing of reproduction within rivers, which can be delayed by several weeks during cold years	Ref. 64
Glochidial development	Germany	Growth of encysted glochidia starts with temperature at approximately 5°C on average; growth has linear relationship with effective temperature sum (based on cumulative degree days)	Refs. 61, 65
Glochidial development	Germany	High temperature is a limiting factor for glochidial development	Ref. 66
Glochidial development	Norway	Higher temperatures decrease the duration of the parasitic phase, but juveniles with the longest parasitic phase have better chances (larger size and faster growth rate) to survive their first winter. The temperature dependence of this interaction suggests that climate warming may affect the relationship between pearl mussels and their hosts	Ref. 67
Mortality of juvenile mussels	Germany	Temperature is the most important factor, showing a highly significant positive correlation with mortality of juvenile mussels. Larger individuals have lower mortality than smaller ones but mortality increases with rising water temperature, i.e., a rise in water temperature impairs the chance of a juvenile mussel benefiting from its accelerated growth	Ref. 24
Life cycle	Europe	Decline in host fish stocks due to climate warming is indirectly affect life cycle of mussels	Refs. 17–18

References

1. G.-R. Walther *et al.*, Ecological responses to recent climate change. *Nature* **416**, 389–395 (2002); DOI:10.1038/416389a
2. G. Woodward, D. M. Perkins, L. E. Brown, Climate change and freshwater ecosystems: impacts across multiple levels of organization. *Phil. Trans. R. Soc. B* **365**, 2093–2106 (2010); DOI:10.1098/rstb.2010.0055
3. M. C. Urban *et al.*, Improving the forecast for biodiversity under climate change. *Science* **353**, aad8466 (2016); DOI:10.1126/science.aad8466
4. M. C. Urban, Accelerating extinction risk from climate change. *Science* **348**, 571–573 (2015); DOI:10.1126/science.aaa4984
5. C. Chen, J. K. Hill, R. Ohlemüller, D. B. Roy, C. D. Thomas, Rapid range shifts of species associated with high levels of climate warming. *Science* **333**, 1024–1026 (2011); DOI:10.1126/science.1206432
6. B. R. Schöne, E. Dunca, H. Mutvei, U. Norlund, A 217-year record of summer air temperature reconstructed from freshwater pearl mussels (*M. margarifitera*, Sweden). *Quaternary Sci. Rev.* **23**, 1803–1816 (2004); DOI:10.1016/j.quascirev.2004.02.017
7. M. Dokulil, Impact of climate warming on European inland waters. *Inland Waters* **4**, 27–40 (2013).
8. R. Arora, K. Tockner, M. Venohr, Changing river temperatures in northern Germany: trends and drivers of change. *Hydrol. Process.* **30**, 3084–3096 (2016); DOI:10.1002/hyp.10849
9. J. Heino, R. Virkkala, H. Toivonen, Climate change and freshwater biodiversity: detected patterns, future trends and adaptations in northern regions. *Biol. Rev.* **84**, 39–54 (2009); DOI:10.1111/j.1469-185X.2008.00060.x
10. J. Geist, Integrative freshwater ecology and biodiversity conservation. *Ecol. Indic.* **11**, 1507–1516 (2011); DOI:10.1016/j.ecolind.2011.04.002
11. B. Basarin, T. Lukić, D. Pavić, R. L. Wilby, Trends and multi-annual variability of water temperatures in the river Danube, Serbia. *Hydrol. Process.* **30**, 3315–3329 (2016); DOI:10.1002/hyp.10863
12. K. Tockner, U. Uehlinger, C. T. Robinson, *Rivers of Europe* (Academic Press, Cambridge, 2009).
13. V. Ziuganov, A. Zotin, L. Nezlin, V. Tretiakov, *The freshwater pearl mussels and their relationships with salmonid fish* (VNIRO Publishing House, Moscow, 1994).
14. A. Makhrov *et al.*, Historical geography of pearl harvesting and current status of populations of freshwater pearl mussel *Margaritifera margaritifera* (L.) in the western part of Northern European Russia. *Hydrobiologia* **735**, 149–159 (2014); DOI:10.1007/s10750-013-1546-1
15. J. Tews *et al.*, Animal species diversity driven by habitat heterogeneity/diversity: the importance of keystone structures. *J. Biogeogr.* **31**, 79–92 (2004); DOI:10.1046/j.0305-0270.2003.00994.x
16. J. Geist, Strategies for the conservation of endangered freshwater pearl mussels (*Margaritifera margaritifera* L.): a synthesis of conservation genetics and ecology. *Hydrobiologia* **644**, 69–88 (2010); DOI:10.1007/s10750-010-0190-2
17. P. Cosgrove *et al.*, The status of the freshwater pearl mussel *Margaritifera margaritifera* in Scotland: extent of change since 1990s, threats and management implications. *Biodivers. Conserv.* **25**, 2093-2112 (2016); DOI:10.1007/s10531-016-1180-0

18. L. C. Hastie, P. J. Cosgrove, N. Ellis, M. J. Gaywood, The threat of climate change to freshwater pearl mussel populations. *AMBIO* **32**, 40–46 (2003); DOI:10.1579/0044-7447-32.1.40
19. R. M. B. Santos *et al.*, Impacts of climate change and land-use scenarios on *Margaritifera margaritifera*, an environmental indicator and endangered species. *Sci. Total Environ.* **511**, 477–488 (2015); DOI:10.1016/j.scitotenv.2014.12.090
20. D. Jacobsen, A. M. Milner, L. E. Brown, O. Dangles, Biodiversity under threat in glacier-fed river systems. *Nat. Clim. Change* **2**, 361–364 (2012); DOI:10.1038/nclimate1435
21. M. Lopes-Lima *et al.*, Conservation status of freshwater mussels in Europe: state of the art and future challenges. *Biol. Rev.* **92**, 572–607 (2017); DOI:10.1111/brv.12244
22. G. Bauer, Threats to the freshwater pearl mussel *Margaritifera margaritifera* in central Europe. *Biol. Conserv.* **45**, 239–253 (1988); DOI:10.1016/0006-3207(88)90056-0
23. G. Bauer, The status of the freshwater pearl mussel *Margaritifera margaritifera* L. in the south of its European range. *Biol. Conserv.* **38**, 1–9 (1986); DOI:10.1016/0006-3207(86)90015-7
24. V. Buddensiek, The culture of juvenile freshwater pearl mussels *Margaritifera margaritifera* L. in cages: a contribution to conservation programmes and the knowledge of habitat requirements. *Biol. Conserv.* **74**, 33–40 (1995); DOI:10.1016/0006-3207(95)00012-S
25. R. Sousa *et al.*, Conservation status of the freshwater pearl mussel *Margaritifera margaritifera* in Portugal. *Limnologica* **50**, 4–10 (2015); DOI:10.1016/j.limno.2014.07.004
26. C. Gumpinger, W. Heinisch, J. Moser, T. Ofenböck, C. Stundner, *Die Flussperlmuschel in Österreich* (Umweltbundesamt GmbH., Wien, 2002).
27. E. Quinlan *et al.*, A review of the physical habitat requirements and research priorities needed to underpin conservation of the endangered freshwater pearl mussel *Margaritifera margaritifera*. *Aquat. Conserv.* **25**, 107–124 (2015); DOI:10.1002/aqc.2484
28. P. J. Colby, G. R. Spangler, D. A. Hurley, A. M. McCombie, Effects of eutrophication on salmonid communities in oligotrophic lakes. *J. Fish. Res. Board Can.* **29,** 975–983 (1972).
29. A. Zieritz, D. C. Aldridge, Identification of ecophenotypic trends within three European freshwater mussel species (Bivalvia: Unionoida) using traditional and modern morphometric techniques. *Biol. J. Linn. Soc.* **98**, 814–825 (2009); DOI:10.1111/j.1095-8312.2009.01329.x
30. G. Bauer, Variation in the life span and size of the freshwater pearl mussel. *J. Anim. Ecol.* **61**, 425–436 (1992); DOI:10.2307/5333
31. S. Helama, I. Valovirta, Shell morphometry, pre-mortal taphonomy and ontogeny-related growth characteristics of freshwater pearl mussel in northern Finland. *Ann. Zool. Fenn.* **44**, 285–302 (2007). Stable URL: http://www.jstor.org/stable/23736772
32. G. A. Weyhenmeyer, Y. T. Prairie, L. J. Tranvik, Browning of boreal freshwaters coupled to carbon-iron interactions along the aquatic continuum. *PLoS ONE* **9**, e88104 (2014); DOI: 10.1371/journal.pone.0088104
33. J. J. Wiens Climate-related local extinctions are already widespread among plant and animal species. *PLoS Biology* **14,** e2001104 (2016).
34. R. R. Sokal, F. J. Rohlf, *Introduction to Biostatistics, 2nd Edition* (Dover Publications, New York, 2009).
35. H. Söderberg, Flodpärlmussla – vad behöver vi göra för att rädda arten? *Karlstad University Studies* **15**, 5–8 (2006).
36. A. Zieritz, D. C. Aldridge, Sexual, habitat-constrained and parasite-induced dimorphism in the shell of a freshwater mussel (*Anodonta anatina*, Unionidae). *J. Morphol.* **272**, 1365–1375 (2011); DOI:10.1002/jmor.10990

37. M. J. Crawley, *Statistical computing, an introduction to data analysis using S-plus* (John Wiley and Sons Ltd., Chichester, 2002).

38. I. Harris, P. D. Jones, T. J. Osborn, D. H. Lister, Updated high-resolution grids of monthly climatic observations – the CRU TS 3.10 Dataset. *Int. J. Climatol.* **34**, 623–642. (2014); DOI:10.1002/joc.3711

39. R. Kohavi, A study of cross-validation and bootstrap for accuracy estimation and model selection. *Ijcai* **14,** 1137–1145 (1995).

40. C. Tofallis, A better measure of relative prediction accuracy for model selection and model estimation. *J. Oper. Res. Soc.* **66,** 1352–1362 (2014).

41. M. J. Crawley, *The R book* (John Wiley and Sons Ltd., Chichester, 2002).

42. D. P. Van Vuuren *et al.*, The representative concentration pathways: an overview. *Climatic Change* **109**, 5–31 (2011); DOI:10.1007/s10584-011-0148-z

43. R. J. Hijmans, S. E. Cameron, J. L. Parra, P. G. Jones, A. Jarvis, Very high resolution interpolated climate surfaces for global land areas. *Int. J. Clim.* **25**, 1965–1978 (2005); DOI:10.1002/joc.1276

44. M. N. Semenova, L. A. Karpycheva, B. B. Voloshenko, V. F. Bugaev, Comparative analysis of growth rates in the European pearl mussel *Margaritifera margaritifera* (Bivalvia, Margaritiferidae) [In Russian]. *Zool. Zh.* **71**, 19–27. (1992).

45. V. Ziuganov *et al.*, Life span variation of the freshwater pearl shell: a model species for testing longevity mechanisms in animals. *AMBIO* **28**, 102–105 (2000); DOI:10.1579/0044-7447-29.2.102

46. O. Moog, H. Nesemann, T. Ofenböck, C. Stundner, *Grundlagen zum Schutz der Flussperlmuschel in Österreich* (Institut für Wasservorsorge, Gewässergüte und Fischerei, Universität für Bodenkultur, Wien, 1993).

47. D. Csar, C. Scheder, C. Gumpinger, The freshwater pearl mussel in Austria – current status and prospects for the future. *Karlstad University Studies* **40**, 61–68 (2012).

48. M. Jung, C. Scheder, C. Gumpinger, J. Waringer, Habitat traits, population structure and host specificity of the freshwater pearl mussel *Margaritifera margaritifera* in the Waldaist River (Upper Austria). *Biologia* **68**, 922–931 (2013); DOI:10.2478/s11756-013-0244-9

49. C. Scheder, C. Gumpinger, The freshwater pearl mussel (*Margaritifera margaritifera* Linné, 1758) in Upper Austria – A species threatened with extinction and current measures for its sustained protection. *Rom. J. Biol.-Zool.* **52**, 53–59 (2008).

50. M. Rudzīte, Distribution of the freshwater pearl mussel *Margaritifera margaritifera* (Linnaeus 1758) in Latvia in relation to water quality. *Acta Universitatis Latviensis* **676**, 79–85 (2004).

51. M. Rudzīte, M. Rudzītis, J. Birzaks, A. Poppels, A. Onkele, The freshwater pearl mussel *Margaritifera margaritifera* (Linnaeus 1758) in Latvia - assessment for the survival possibilities. *Schr. Malakozool.* **28**, 17–36 (2015).

52. M. Rudzīte, Assessment of the condition of freshwater pearl mussel *Margaritifera margaritifera* (Linnaeus 1758) populations in Latvia. *Acta Universitatis Latviensis* **691**, 121–128 (2005).

53. A. N. Ostrovsky, I. Y. Popov, Rediscovery of the largest population of the freshwater pearl mussel (*Margaritifera margaritifera*) in the Leningrad oblast (north-west Russia). *Aquat. Conserv.* **21**, 113–121 (2011); DOI:10.1002/aqc.1164

54. I. Y. Popov, A. N. Ostrovsky, Survival and extinction of the southern populations of freshwater pearl mussel *Margaritifera margaritifera* in Russia (Leningradskaya and Novgorodskaya oblast). *Hydrobiologia* **735**, 161–177 (2014); DOI:10.1007/s10750-013-1640-4

55. I. N. Bolotov *et al.*, Influence of historical exploitation and recovery of biological resources on contemporary status of *Margaritifera margaritifera* L. and *Salmo salar* L. populations in Northwestern Russia. *Biol. Bull. Rev.* **2**, 460–447 (2012); DOI:10.1134/S2079086412060035

56. J. Bergengren, J. Törnblom, *Återintroduktion av flodpärlmussla. Uppföljning av utplantering av glochidieinfekterad öring i Hyttkvarnsån* (Världsnaturfonden WWF, 2005).

57. K. Jarl, *Flodpärlmusslan i Marks kommun - hot mot populationen* (Miljökontoret, Marks kommun, 2007).

58. P. Ingvarsson, Flodpärlmussla i Hallands län - en kompletterande inventering. Rapport 2011: 9 (2011); Stable URL: http://www.lansstyrelsen.se.

59. Y. V. Bespalaja, I. N. Bolotov, A. A. Makhrov, State of the population of the European pearl mussel *Margaritifera margaritifera* (L.) (Mollusca, Margaritiferidae) at the northeastern boundary of its range (Solza River, White Sea Basin). *Russ. J. Ecol.* **37**, 222–229 (2007); DOI:10.1134/S1067413607030095

60. E. S. Miguel *et al.*, Growth models and longevity of freshwater pearl mussels (*Margaritifera margaritifera*) in Spain. *Can. J. Zoolog.* **82**, 1370–1379 (2004); DOI:10.1139/z04-113

61. C. Schmidt, R. Vandré, Ten years of experience in the rearing of young freshwater pearl mussels (*Margaritifera margaritifera*). *Aquat. Conserv.* **20**, 735–747 (2010); DOI:10.1002/aqc.1150

62. V. Altnoder, Beobachtungen uber die Biologie von *Margaritifera margaritifera* und über die Okologie ihres Wohnortes. *Arch. Hydrobiol.* **17**, 423–491 (1926).

63. G. Wellmann, Untersuchungen über die Flussperlmuschel (*Margaritifera margaritifera* L.) und ihren Lebensraum in Bachen der Lüneburger Heide. *Zeit. Fisch.* **36**, 489–603 (1939).

64. L. C. Hastie, M. R. Young, Timing of spawning and glochidial release in Scottish freshwater pearl mussel (*Margaritifera margaritifera*) populations. *Freshwater Biol.* **48**, 2107–2117 (2003); DOI:10.1046/j.1365-2427.2003.01153.x

65. T. Eybe, F. Thielen, T. Bohn, B. Sures, Influence of the excystment time on the breeding success of juvenile freshwater pearl mussels (*Margaritifera margaritifera*). *Aquat. Conserv.* **25**, 21–30 (2015); DOI:10.1002/aqc.2471

66. J. Hruška, The freshwater pearl mussel in South Bohemia: evaluation of the effect of temperature on reproduction, growth and age structure of the population. *Arch. Hydrobiol.* **126**, 181–191 (1992).

67. J. Marwaha, K. H. Jensen, P. J. Jakobsen, J. Geist, Duration of the parasitic phase determines subsequent performance in juvenile freshwater pearl mussels (*Margaritifera margaritifera*). *Ecol. Evol.* **7**, 1375–1383 (2017); DOI:10.1002/ece3.2740
